# Nanoarchitectonics of Bactericidal Coatings Based on CaCO_3_–Nanosilver Hybrids

**DOI:** 10.1021/acsabm.3c01228

**Published:** 2024-05-09

**Authors:** Ana M. Ferreira, Anna S. Vikulina, Laura Bowker, John A. Hunt, Michael Loughlin, Valeria Puddu, Dmitry Volodkin

**Affiliations:** ^†^School of Science and Technology, Department of Chemistry and Forensics, ^§^School of Science and Technology, Department of Biosciences, Nottingham Trent University, Clifton Lane, Nottingham NG11 8NS, U.K.; ‡ Bavarian Polymer Institute, Friedrich-Alexander-Universität Erlangen-Nürnberg (FAU), Dr.-Mack-Straße, 77, 90762 Fürth, Germany

**Keywords:** calcium carbonate, polyvinylpyrrolidone, mucin, vaterite, antimicrobial

## Abstract

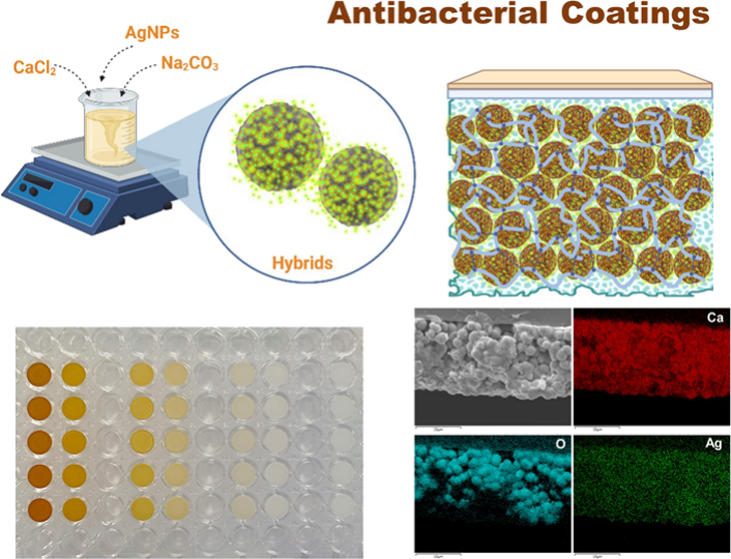

Antimicrobial coatings provide protection against microbes colonization on surfaces. This can prevent the stabilization and proliferation of microorganisms. The ever-increasing levels of microbial resistance to antimicrobials are urging the development of alternative types of compounds that are potent across broad spectra of microorganisms and target different pathways. This will help to slow down the development of resistance and ideally halt it. The development of composite antimicrobial coatings (CACs) that can host and protect various antimicrobial agents and release them on demand is an approach to address this urgent need. In this work, new CACs based on microsized hybrids of calcium carbonate (CaCO_3_) and silver nanoparticles (AgNPs) were designed using a drop-casting technique. Polyvinylpyrrolidone and mucin were used as additives. The CaCO_3_/AgNPs hybrids contributed to endowing colloidal stability to the AgNPs and controlling their release, thereby ensuring the antibacterial activity of the coatings. Moreover, the additives PVP and mucin served as a matrix to (i) control the distribution of the hybrids, (ii) ensure mechanical integrity, and (iii) prevent the undesired release of AgNPs. Scanning electron microscopy (SEM), X-ray diffraction (XRD), and Fourier transform infrared (FTIR) techniques were used to characterize the 15 μm thick CAC. The antibacterial activity was determined against *Escherichia coli*, methicillin-resistant *Staphylococcus aureus* (MRSA), and *Pseudomonas aeruginosa*, three bacteria responsible for many healthcare infections. Antibacterial performance of the hybrids was demonstrated at concentrations between 15 and 30 μg/cm^2^. Unloaded CaCO_3_ also presented bactericidal properties against MRSA. *In vitro* cytotoxicity tests demonstrated that the hybrids at bactericidal concentrations did not affect human dermal fibroblasts and human mesenchymal stem cell viability. In conclusion, this work presents a simple approach for the design and testing of advanced multicomponent and functional antimicrobial coatings that can protect active agents and release them on demand.

## 1 Introduction

Antimicrobial materials are crucial in reducing the spread of pathogenic microorganisms from contaminated surfaces. Their importance is most significant in settings where there is the risk of infecting already sick or injured people, such as in hospitals and other healthcare settings. In this often highly populated busy environments, the spread of dangerous microorganisms through contaminated surfaces is a real risk to patients.^1^ Different antimicrobial agents, predominantly metals and metal oxides, including silver, copper, zinc, and titanium dioxide, have been incorporated into antibacterial coatings, with silver being the most commonly used.^2,3^ Silver nanoparticles (AgNPs) and silver salts present a broad-spectrum antimicrobial activity; however, the release of silver into the environment raises concerns about its toxicity on living organisms.^4−6^ Moreover, AgNPs present variable stability when exposed to light, salts, and biomolecules, which can decrease their effectiveness.

Different platforms have been used to immobilize and control the release of AgNPs, including calcium carbonate (CaCO_3_) vaterite.^4^ Vaterite crystals are metastable polymorphs of CaCO_3_ that, due to their porous structure, can accommodate AgNPs, protecting them from external factors.^4^ Moreover, the dissolution of vaterite at acidic pH makes these carriers ideal for releasing AgNPs in the acidic microenvironments of acute inflammation sites or in the core of biofilms.^7^

The mechanism of AgNP release from vaterite has been previously studied and is based on either pH-mediated dissolution of CaCO_3_ or vaterite recrystallization into calcite in aqueous solutions. The release of AgNPs is also controlled by the medium composition and the affinity to AgNPs.^4^

The production of coatings entails the use of polymers that promote the coating adhesion to the substrate, either through mechanical interlocking, electrostatic interactions, polymer chain entanglement, or intermolecular bonding like van der Waals forces and covalent and hydrogen bonds.^8−10^ Polyvinylpyrrolidone (PVP) is a nontoxic, water-soluble polymer with excellent binding and film-forming properties.^11^ PVP also presents a good affinity for both hydrophilic and hydrophobic compounds due to its hydrophilic pyrrolidone moiety and hydrophobic alkyl groups.^12,13^ Due to these properties, PVP has already been used to produce coatings and adhesives.^12^

An important parameter when developing coatings with vaterite is selecting polymers that can also halt the recrystallization of vaterite into calcite and therefore providing for controlled and sustained release.

Mucins are large bioactive extracellular glycoproteins mainly composed of carbohydrates, with the main functions being lubrication, hydration, working as a barrier, and regulation of biological responses.^14^ Adding to that, mucins present good adhesive properties, and their bulky structure with rich chemistry can work as a stabilizing agent for vaterite by suppressing the diffusion of ions from the surface of the crystals.^15,16^ Moreover, materials coated with some mucins present improved compatibility and antifouling properties.^14^

In this work, hybrids composed of vaterite and AgNPs (CaCO_3_/AgNPs) were synthesized and used in the production of antibacterial coatings. A simple method was applied to devise the coatings, and a formulation composed of hybrids, PVP, and mucin developed. The drop-casting technique was selected to coat polystyrene due to the simplicity of the method and low wastage of sample compared with other methods like spin coating.^17^ Moreover, it is closely aligned with relevant coating processes and facilitates the coating of substrates with varying geometries like fibers and foams.^18−20^

The coatings were prepared on 96-well plates, which minimized the quantity of sample required for their production and then characterized using scanning electron microscopy (SEM), X-ray diffraction (XRD), and Fourier transform infrared (FTIR). The release of AgNPs was also determined, as this is an important parameter to evaluate the role of the coatings in preventing the premature release of AgNPs. The antibacterial activity of the coatings was assessed against *Escherichia coli* (*E. coli*), methicillin-resistant *Staphylococcus aureus* (MRSA), and *Pseudomonas aeruginosa* (*P. aeruginosa*). The influence of the additives PVP and mucin on the antibacterial activity was also studied, as well as the *in vitro* cytotoxicity of the hybrids at bactericidal concentrations against human cells.

Overall, this work determined the potential of CaCO_3_/AgNP hybrids as active components for the production of surfaces with a strong antibacterial activity. A simple approach to rapidly produce coatings and test their antibacterial activity is proposed. This opens new routes to accelerate the development of novel antibacterial coatings, crucial to reduce the spread of pathogenic bacteria through contact and residence on surfaces.

## 2 Materials and Methods

### 2.1 Materials

Sodium borohydride (NaBH_4_, ≥99% pure), polyvinylpyrrolidone 40 kDa, polyvinylpyrrolidone 150–300 kDa (PVP), Mueller Hinton broth Sigma 70192 (MHB), Mueller Hinton agar Sigma 70191 (MHA), phosphate buffer saline (PBS) tablets, Calbiochem mucin from the bovine submaxillary gland, trypsin-EDTA solution, heat-inactivated fetal bovine serum (FBS), and ICP-MS standard TraceCERT 1 ppm of silver in 2% nitric acid were obtained from Sigma-Aldrich (Steinheim, Germany). Mesenchymal stem cell growth medium Bullet Kit was purchased from Lonza (Walkersville, MD). Silver nitrate (AgNO_3_, ≥99% pure), calcium chloride dihydrate (CaCl_2_·2H_2_O, ≥99% pure), sodium carbonate (Na_2_CO_3_, ≥99.5%), tris buffer saline 10X solution (TBS), 99% ethanol, hexamethyldisiloxane 98% (HMDS), dimethyl sulfoxide, high-purity resazurin sodium salt (80% min), 3-(4,5-dimethylthiazol-2-yl)-2,5-diphenyltetrazolium bromide (MTT), Gibco penicillin–streptomycin (10,000 U·mL^–1^), Gibco low glucose-GlutaMAX supplement Dulbecco’s modified Eagle’s medium (DMEM), sodium hydroxide pellets (NaOH, ≥98% pure), glacial acetic acid (CH_3_COOH, ≥99.7% pure), 70% nitric acid (HNO_3_, analytical grade), and 37% hydrochloric acid (HCl, analytical grade) were obtained from Fisher Scientific (Loughborough, United Kingdom).

### 2.2 Methods

#### 2.2.1 Synthesis of AgNPs

AgNPs were synthesized via a modified chemical reduction methodology adapted from Izak-Nau et al.^21^ Briefly, freshly prepared NaBH_4_ (40 mL, 0.01 M) was added dropwise (*ca*. 2 drops/s) at room temperature and under stirring (850 rpm) to AgNO_3_ (2 mL, 0.1 M) previously mixed with Milli-Q water (158 mL) and PVP. The final capping agent concentration was 0.38 mg·mL^–1^. After synthesis, the AgNPs were filtered and then washed with Milli-Q water by centrifugation (5000*g* for 30 min) using Pierce Protein Concentrators PES with a 50 K molecular weight cutoff membrane (Thermo Fisher Scientific, Germering, Germany). The particles were then resuspended in deionized water, and the silver concentration determined by inductively coupled plasma mass spectrometry (ICP-MS).

##### 2.2.1.1 TEM Analysis of the Silver Nanoparticles

A AgNP–PVP stock colloidal dispersion was diluted with Milli-Q water, and then 7 μL was added on top of a holey carbon film copper grid (Agar Scientific Ltd., U.K.) and left drying overnight before analysis on a JEM-2100 Plus transmission electron microscope (Jeol, Japan), using an operating voltage of 200 kV. The diameter of 300 particles was measured to estimate the particle size and distribution using the ImageJ software (NIH, USA).

#### 2.2.2 Synthesis of Bare CaCO_3_

Bare vaterite CaCO_3_ was synthesized based on the work of Volodkin et al.^22^ Briefly, CaCl_2_·2H_2_O (150 mM) was mixed with an equal volume of TBS 6× and Milli-Q water under intense magnetic stirring (1400 rpm). Then, Na_2_CO_3_ (50 mM) was added, and the stirring continued for 30 s. The suspension was poured into a tube and left for 10 min to allow the crystals to grow. After that, the suspension was centrifuged at 3000*g* for 5 min, washed with 25 mL of Milli-Q water via resuspension, centrifuged for another 5 min, and resuspended in 300 μL of 99% ethanol. The crystals were then dried at 80 °C for 40 min. The initial molar ratio between CaCl_2_·2H_2_O and Na_2_CO_3_ was 1:1. All of the syntheses were performed at room temperature, and the reagent solutions were prefiltered with a 0.2 μm syringe-tip filter (Fisherbrand, Loughborough, United Kingdom).

#### 2.2.3 Synthesis of CaCO_3_/AgNP Hybrids

AgNPs were loaded into CaCO_3_ crystals by cosynthesis. Briefly, CaCl_2_·2H_2_O (150 mM) was mixed with an equal volume of TBS 6×, and then AgNPs and Milli-Q water were added under intense magnetic stirring (1400 rpm). A few seconds later, Na_2_CO_3_ (50 mM) was added, and the stirring continued for 30 s. Then, the suspension was poured into a tube and left for 10 min to allow the crystals to grow. After that, the dispersion was centrifuged at 3000*g* for 5 min, washed with 25 mL of Milli-Q water via resuspension, centrifuged for another 5 min, and resuspended in 300 μL of 99% ethanol. The crystals were then dried at 80 °C for 40 min. The molar ratio between CaCl_2_·2H_2_O and Na_2_CO_3_ was 1:1, and the mass ratio between AgNPs and CaCO_3_ was 30 mg·g^–1^. The synthesis was performed at room temperature, and the reagent solutions were prefiltered with a 0.2 μm syringe-tip filter (Fisherbrand, Loughborough, United Kingdom). 450 particles were analyzed to estimate the particle size and distribution using the ImageJ software (NIH, USA). The mass of silver loaded into CaCO_3_ (mg·g^–1^) was determined by ICP-MS, and the morphology of the crystals was analyzed by SEM after being coated with a 5 nm layer of gold.

#### 2.2.4 Silver Concentration Measurement by ICP-MS

AgNP colloidal dispersions and CaCO_3_/AgNP hybrids were digested with a fresh mixture of one part of 70% HNO_3_ and three parts of 37% HCl (v/v) to ensure the formation of soluble silver chloride complexes (AgCl_*x*_^(*x*–1)–^) instead of insoluble AgCl salts. All of the digested samples presented a concentration of silver lower than 10 μg mL^–1^ and a HCl content higher than 10% (v/v). The samples were digested at room temperature in the dark for over 1 h, and then 7–14 μL of the digested samples was diluted with 1 mL of 2% HNO_3_ before analysis. A calibration curve was obtained for each independent ICP analysis with silver concentrations ranging between 3 and 800 μg·L^–1^. The coefficient of determination of the standard calibration curve was always superior to 0.998. Both ^107^Ag and ^109^Ag isotopes were quantified, and ^109^Ag signal was used to determine the content of silver in all of the samples. Steps were implemented to reduce exposure to light in order to minimize photoreduction, like keeping the samples in the dark when possible and handling them without direct exposure to light.

#### 2.2.5 Coating Production

The drop-casting technique was selected to coat flat-bottom hydrophilic polystyrene wells in a 96-well microplate (SARSTEDT, Germany) due to its straightforwardness and lower wastage of hybrids. The formulation and coating steps were optimized to produce uniform surfaces and prevent the recrystallization of the hybrids.

##### 2.2.5.1 Method Optimization

Dispersions of CaCO_3_/AgNP hybrids in Milli-Q water, with and without PVP, were prepared by mixing all of the components at 2000 rpm for 5 min in a ThermoMixer C with a SmartBlock (Eppendorf, Germany), followed by sonication at 30 Hz, power 30%, for 5 min in a Fisherbrand Transsonic TI-H-10 ultrasonic bath (Fisher Scientific, UK) and a final mixing for 3 min. After that, 60 or 90 μL of the dispersions were poured on the wells of a 96-well microplate and dried in the oven (Heratherm oven, Thermo Scientific) at 50 and 37 °C for 2 and 20 h, respectively, or at 80 °C for 2 h. The concentrations of CaCO_3_/AgNP hybrids and PVP in the coating dispersions were 6 and 8 mg·mL^–1^, respectively. After drying the coatings, the adhesion to the wells was tested by scraping the formed coatings with a spatula. The coatings were also analyzed under the microscope (Life Technologies EVOS FL, Invitrogen) to study the effect of the coating production steps on the polymorphism of the hybrids.

##### 2.2.5.2 Optimized Method

Due to the recrystallization of the hybrids during the coating production, mucin was added to the formulation as it can halt the transformation of vaterite into calcite.^23^ Briefly, CaCO_3_/AgNP hybrids were mixed with a solution of mucin for 10 min at 2000 rpm, and then PVP and Milli-Q water were added and mixed at 2000 rpm for another 5 min. After that, the dispersion was sonicated for 5 min at 35 Hz, 30% power, to break apart any clusters of hybrids, and then mixed for 3 min before being poured into the wells of a 96-well microplate (90 μL/well). Formulations with different concentrations of hybrids were prepared by replacing the hybrids with equal quantities of bare CaCO_3_ vaterite. Regardless of the CaCO_3_/AgNP hybrid concentration, the concentration of PVP and mucin were kept constant, *i.e*., at 8 and 1 mg·mL^–1^, respectively, as well as the total concentration of bare CaCO_3_ and hybrids (6 mg·mL^–1^). The coating layout is depicted in Figure S1. This layout was selected because of allowing to rapidly test the antibacterial activity of the coatings at different concentrations of hybrids and respective replicates, eliminating the need for additional steps or a new layout.

##### 2.2.5.3 Characterization of the Coatings

**SEM:** the coatings were cut out from the microplates with a laser cutter and then mounted on stubs by using double-sided carbon tape. To determine the thickness of the coatings, cross sections were also analyzed. All of the samples were coated with a 5 nm thick layer of gold (Quorum Q150R ES, U.K.) and analyzed on a field emission electron microscope (JEOL, JSM-7100f, Tokyo, Japan) with a secondary electron detector and an acceleration voltage of 10.0 kV. Three independent samples were analyzed using the ImageJ software (NIH) to determine the average thickness of the coatings.

**SEM coupled with energy-dispersive X-ray spectroscopy (SEM–EDS):** samples of the cross-sectioned coatings were mounted on stubs using double-sided carbon tape and then coated with a 5 nm thick layer of gold. An accelerating voltage of 10 kV and a working distance of 10 mm were used. The probe current was optimized to give a dead time of around 45%.

**Fourier transform infrared (FTIR) spectroscopy:** previously formed coatings were humidified with Milli-Q water, and portions were scratched out and placed on the FTIR spectrometer (Spectrum Two FTIR spectrometer, PerkinElmer, Uberlingen, Germany). Before analysis, the sample was quickly dried with an air gun, until no water peaks were detectable. Mucin, PVP, and CaCO_3_/AgNPs were also analyzed. The analysis settings were 32 scans per sample between 500 and 4000 cm^–1^ with a resolution of 4 cm^–1^. The attenuated total reflectance (ATR) technique was used in all of the measurements.

**X-ray diffraction (XRD):** the crystallinity of the coatings was analyzed with the SmartLab SE X-ray diffraction system from Rigaku Co. Ltd. (Tokyo, Japan) with a Kβ filter for copper (λ = 0.1392 nm). Samples were scanned with the θ/2θ scan axis. The scan range varied between 20 and 80°, and the scan mode and speed were 1D and 5°/min, respectively.

### 2.3 Analysis of the Release of AgNPs from the Coatings

The release of AgNPs from the coating only composed of hybrids, and the coating composed of hybrids, mucin, and PVP, was assessed in MHB and TBS (unless otherwise specified TBS is TBS 1×, i.e., 10 times diluted from TBS 10×). MHB medium was chosen because it was used in all of the bacterial assays in this work. This medium can also simulate scenarios in which a coating is exposed to environments abundant in energy sources for bacteria. Additionally, it is composed of polymers that can interact with the composite components. Conversely, TBS was selected as a control due to its simple composition, comprising only tris and salts at low concentrations. It can also mimic environments with a negligible composition that have a limited impact on the coating or bacteria.

The release study comprised adding 200 μL of TBS or MHB to the coated wells and then incubating them at 37 °C overnight (ca. 19 h). The next day, TBS and MHB were aspirated from the wells and centrifuged at 2350*g* for 5 min to settle any hybrids that dislodged during incubation or aspiration of the supernatant. After that, the supernatant was analyzed by UV–vis spectrophotometry (NanoDrop One spectrophotometer, Thermo Scientific) to detect released AgNPs. TBS and MHB were used as the blanks. For comparison, the exact content of AgNPs on the coatings was dispersed in the same volume of TBS and MHB (200 μL) and analyzed on the UV–vis spectrophotometer. All samples were diluted with Milli-Q water until the absorbance was below one. The data was normalized for a better comparison between the samples and the dilution factors taken into consideration. The coatings were also analyzed under the microscope to investigate the effect of MHB and TBS on the stability of the hybrids.

### 2.4 Assessment of the Antibacterial Activity

The antibacterial activity of coatings with different concentrations of CaCO_3_/AgNP hybrids was determined against *E. coli* O157:H7 (*E. coli*), methicillin-resistant *Staphylococcus aureus* (MRSA), and *P. aeruginosa* PA01 (*P. aeruginosa*). The *E. coli*, MRSA, and *P. aeruginosa* isolates were obtained from the American Type Culture Collection (ATCC 43888), National Collection of Type Cultures (NCTC 12493), and Nottingham Trent University Collections (NTUCC 876), respectively.

#### 2.4.1 Inoculum Preparation

The bacterial isolates were streaked onto MHA plates and incubated at 37 °C for 18–24 h. For each bacterium, three to four isolated colonies of the same morphological appearance were transferred into a tube containing 5 mL of MHB and then incubated for 18–24 h in a shaker at 35 °C and 225 rpm. Overnight cultures were diluted to 5 × 10^5^ CFU/mL with MHB immediately before incubation with the coatings.

#### 2.4.2 Bacterial Viability after Contact with the Coated Surfaces

The antibacterial activity of the coatings only composed of CaCO_3_/AgNP hybrids (coating A), and the coatings composed of CaCO_3_/AgNP hybrids, PVP, and mucin (coating B), was tested. The final concentration of hybrids was equivalent between the two coating formulations and ranged between 7 and 1862 μ/cm^2^. The assay conditions were selected and optimized based on the reports of Minor et al., García-Cañas et al., and Bittner et al.^24−26^ and comprised the measurement of the bacterial viability using the resazurin reduction assay. Before testing the coatings, the sensitivity of the resazurin reduction assay to different concentrations of viable cells of *E. coli*, MRSA, and *P. aeruginosa* was tested. Briefly, overnight cultures were serially diluted with PBS and then 150 μL of the bacterial suspension mixed with 30 μL of resazurin (0.15 mg·mL^–1^ in PBS), followed by incubation at 37 °C for 2–3 h, after which the fluorescence was measured using a 560 nm excitation/590 nm emission filter set (Cytation 3, BioTek, Vermont). As shown in Figure S2, the fluorescence emitted by resorufin (the product of resazurin reduction by the bacteria) was proportional to the concentration of the bacteria, with *E. coli* and MRSA presenting better correlation coefficients, 0.9838 and 0.9845, respectively, than *P. aeruginosa* (0.9641). Considering the sensitivity of the resazurin assay to the concentration of viable cells, the antibacterial activity of the coatings was tested using this method. Briefly, 20 μL of *E. coli*, MRSA, or *P. aeruginosa*, previously diluted with MHB to 5.0 × 10^5^ CFU·mL^–1^, was added to each well. The final bacterial inoculum density was approximately 3.4 × 10^4^ CFU/cm^2^. The microplates were incubated at 37 °C for 20 h. After that, 200 μL of PBS was added to the wells, followed by 30 μL of resazurin (0.15 mg·mL^–1^ in PBS). The microplates were then incubated at 37 °C for 2.5 h, and then 100 μL of each well was transferred to a new microplate to measure the fluorescence using a 560 nm excitation/590 nm emission filter set. Coated wells were also incubated with an equal volume of sterile MHB to check for any potential bacterial contamination or unwanted reduction of resazurin. These controls were used as blanks and repeated for all of the tested concentrations. The sterility of the MHB medium was controlled by incubating this in uncoated wells under the same conditions. Coatings of bare CaCO_3_ with mucin and PVP and coatings of just bare CaCO_3_, mucin or PVP were also prepared and tested against the bacteria under the same conditions. All of the microplates were sterilized with ethanol (70% v/v) and then dried at 80 °C for 2 h prior to the antibacterial assay. The microplate layout is presented in Figure S1. Each tested condition included four or five replicates.

To confirm the complete bactericidal activity of coating B, 10 μL of the suspension from the wells without detectable bacterial growth was plated onto MHA plates. The agar plates were incubated at 37 °C for 24 h and then read visually. Bacterial growth controls were included in each experiment and consisted of pouring 20 μL of bacterial suspension (5 × 10^5^ CFU/mL) into the uncoated wells followed by incubation at the same conditions. The sterility of the MHB medium was controlled by incubating this under the same conditions on uncoated wells.

#### 2.4.3 SEM of the Bacteria after Contact with the Coatings

Polystyrene discs coated with the hybrids, PVP, and mucin were placed into a 12-well microplate and then 20 μL of *E. coli*, MRSA, or *P. aeruginosa* suspension, previously diluted with MHB to 5.0 × 10^5^ CFU·mL^–1^, poured on top. The concentration of CaCO_3_/AgNPs-PVP hybrids was 29 μg/cm^2^, and the final bacterial inoculum density was approximately 3.4 × 10^4^ CFU/cm^2^. The coated discs were then incubated at 37 °C for 2 h. After that, 1.5 mL of formalin (4% formaldehyde) was added to the wells and left for 10 min to fix the bacteria. Formalin was then removed, and the discs were washed three times with Milli-Q water. After washing, the coatings were dehydrated in graded ethanol solutions (50, 60, 70, 80, 90, and 100%), 5 min per concentration. After dehydration, the samples were infiltrated with HMDS (5 min, twice) to further enhance the drying without the risk of the bacterial cells collapsing. The samples were left drying overnight inside a fume hood for complete evaporation of the HMDS, and then mounted on stubs and coated with a 5 nm layer of gold before analysis. An accelerating voltage of 10 kV and a working distance of 10 mm were selected for SEM imaging.

### 2.5 *In Vitro* Cytotoxicity Assessment

The cytotoxicity of the hybrids was tested on two cell lines, normal human dermal fibroblasts (NHDFs) and human mesenchymal stem cells (hMSCs), acquired from the ATCC and Lonza collections, respectively.

#### 2.5.1 Cell Culture

The NHDFs were cultured in DMEM medium supplemented with 10% FBS and 1% penicillin–streptomycin. The hMSCs were cultured in mesenchymal stem cell growth medium supplemented with mesenchymal stem cell basal medium, SingleQuots supplements, and growth factors. The cells were maintained at 37 °C in a humidified incubator and an atmosphere of 5% CO_2_.

#### 2.5.2 Cell Viability Assay

The MTT reduction assay was selected to study the cytotoxicity of CACO_3_/AgNP hybrids, bare CaCO_3_, and AgNPs. Briefly, the cells were seeded into 96-well plates at a density of approximately 24,000 and 7500 cells/well for the NHDFs and hMSCs, respectively. The final volume was 150 μL per well. The cells were incubated for 18 h before treatment to allow their adherence to the plate. After that, the media was gently aspirated and replaced with fresh media with CaCO_3_/AgNP hybrids, bare CaCO_3_, or AgNPs. The final volume was 200 μL, and the concentration of CaCO_3_/AgNP hybrids and bare CaCO_3_ was 29 μg/cm^2^. The concentration of AgNPs was 0.87 μg/cm^2^, i.e., = equivalent to the amount of AgNPs loaded into the hybrids. The cells were incubated at 37 °C in a humidified incubator and an atmosphere of 5% CO_2_ for 24 h. After the incubation period, 14 μL of MTT in PBS (5 mg·mL^–1^) was added to the wells to a final concentration of 0.3 mg·mL^–1^. Subsequently, the cells were incubated at 37 °C for 2 h. After incubation, the cell culture media was gently aspirated, and the formed formazan crystals were dissolved with 200 μL of dimethyl sulfoxide. The absorbance was measured at 550 and 620 nm in a multiwell plate reader (Cytation 3, BioTek, Vermont). To reduce the effect of interferences from the media and crystals, the final absorbance (Abs_final_) was calculated with the following equation
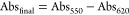
1where Abs_550_ and Abs_620_ represent the absorbance at 550 and 620 nm, respectively.

Each experiment was carried out in triplicate or quadruplicate.

## 3 Results and Discussion

### 3.1 Coating Design

**Figure 1:**
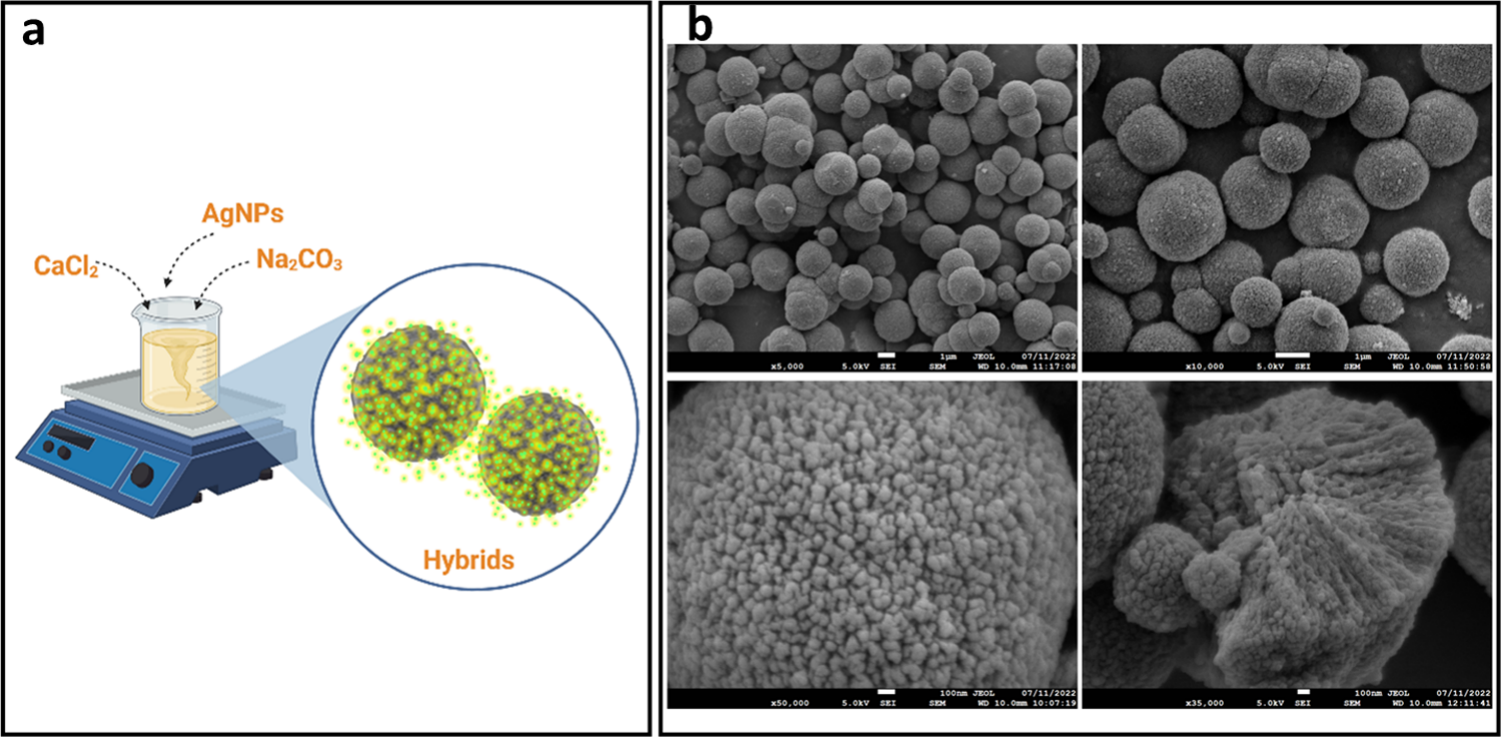
Schematic representation of the synthesis of CaCO_3_/AgNP hybrid (a) and SEM images of the CaCO_3_/AgNP hybrids produced (b). The scale bar represents 1 μm in the top images, and 100 nm in the bottom images, respectively. Scheme a was created with BioRender.com.

One of the aims of this work was to develop a simple method to produce coatings composed of CaCO_3_/AgNP hybrids and determine their potential as active ingredients for antimicrobial coatings. The hybrids used for the production of the coatings were synthesized via coprecipitation of vaterite in the presence of AgNPs with an average diameter of 14 nm (Figures 1a and S3). The formed hybrids had an average diameter of 2.3 ± 0.8 μm. The silver content was 2.98 ± 0.13% (w/w), which was determined by ICP-MS. Figure 1b presents the morphology of the CaCO_3_/AgNP hybrids, which matches the typical features of vaterite crystals, *i.e*., spherically shaped crystals composed of nanocrystallites.

The drop-casting technique was selected to coat the bottom of flat polystyrene wells in a 96-well microplate. This method was selected due to its simplicity and low wastage of sample in comparison with other techniques such as spin coating. Polystyrene microplates were chosen as the coating substrate, as they offer the possibility to rapidly produce different coatings and present a simple layout to run antibacterial tests. It also offers other advantages, such as reproducibility, simplicity, and high output. In one single microplate, it is possible to determine the antibacterial activity of different coatings and corresponding replicates, without requiring a new setup or layout. To the best of our knowledge, this is the first report of this approach.

While the drop-casting technique offers ideal simplicity, it also presents drawbacks, including the development of uneven surfaces, with the formation of the so-called “coffee ring” being common. These rings form due to differences between the solvent evaporation rates in the periphery and center of the “drop”, which promotes the flow of more solvent and solute to the periphery and consequent concentration of nonvolatile components on the edges.^27^ An effect called “coffee-eye” can also occur when drying coatings at temperatures above 40 °C on substrates with low conductivity, like glass and polystyrene.^28^ This pattern forms due to the Marangoni flow, which is created by temperature differences between the droplet edge and the apex.^28^ This effect creates a surface tension gradient, which drives the particles inward and promotes their accumulation in the center of the droplet.^28^ Adding to the complexity of the coating formation, the hybrids present unique challenges, as they can recrystallize in solution into calcite and partially release AgNPs.^4^

Figure S4 presents the coatings produced with two different volumes, 60 and 90 μL, of CaCO_3_ hybrid dispersions in water, with and without PVP as an additive. The coatings produced with the lowest volume (60 μL) presented an irregular distribution of particles, which can be explained by the insufficient number of crystals added, and an outward flow of the hybrids during the drying step. When the dispersion volume added to the wells increased to 90 μL, the well surface became fully coated. Nonetheless, the coating components concentrated in the center of the wells because of the ″coffee-eye” effect. This was more attenuated in the formulation with PVP, as it increased the viscosity and wettability of the dispersion, resulting in more uniform coatings.

The adhesion of the coatings to the wells was tested by scraping the bottom of the wells after drying. As shown in Figure S4c, the coatings without PVP presented poor adhesion to the polystyrene wells and were easily detached from the surface. On the other hand, the coatings with PVP were resistant to scraping, showing that PVP worked as a binder, improving the coating adhesion to polystyrene. The adhesion promoted by PVP resulted from the interactions between PVP/hybrids and PVP/substrate, as well as the entanglement of PVP and possible interlocking with the hybrids due to their porous structure.^29,30^

As depicted in Figure S4 (images d and f), regardless of the formulation, the hybrids became unstable during the coating production and transformed into calcite (cubic shape). This occurred due to prolonged exposure of the hybrids to water, primarily during the drying step, which promoted the recrystallization of metastable vaterite to stable calcite. In order to shorten the drying time, the dispersions were dried at 80 °C instead of following the 50/37 °C cycle. The obtained coatings are presented in Figure S5 (images a and b). Regardless of the formulation, all of the coatings presented a “coffee ring” or “coffee-eye”, which was promoted by outflow or inflow forces, respectively. Figure S5 (images c and d) shows that even with reduced exposure to water due to a higher drying temperature, the hybrids still recrystallized to calcite.

To rule out the effect of the formulation pH on the recrystallization rate, as low pH values accelerate the transformation of vaterite into calcite, the pH of the formulation was analyzed with universal indicator tape. As depicted on the insets in Figure S5 (images c and d), the formulations presented a neutral to basic pH, indicating that the formulation pH did not accelerate the recrystallization.

**Figure 2:**
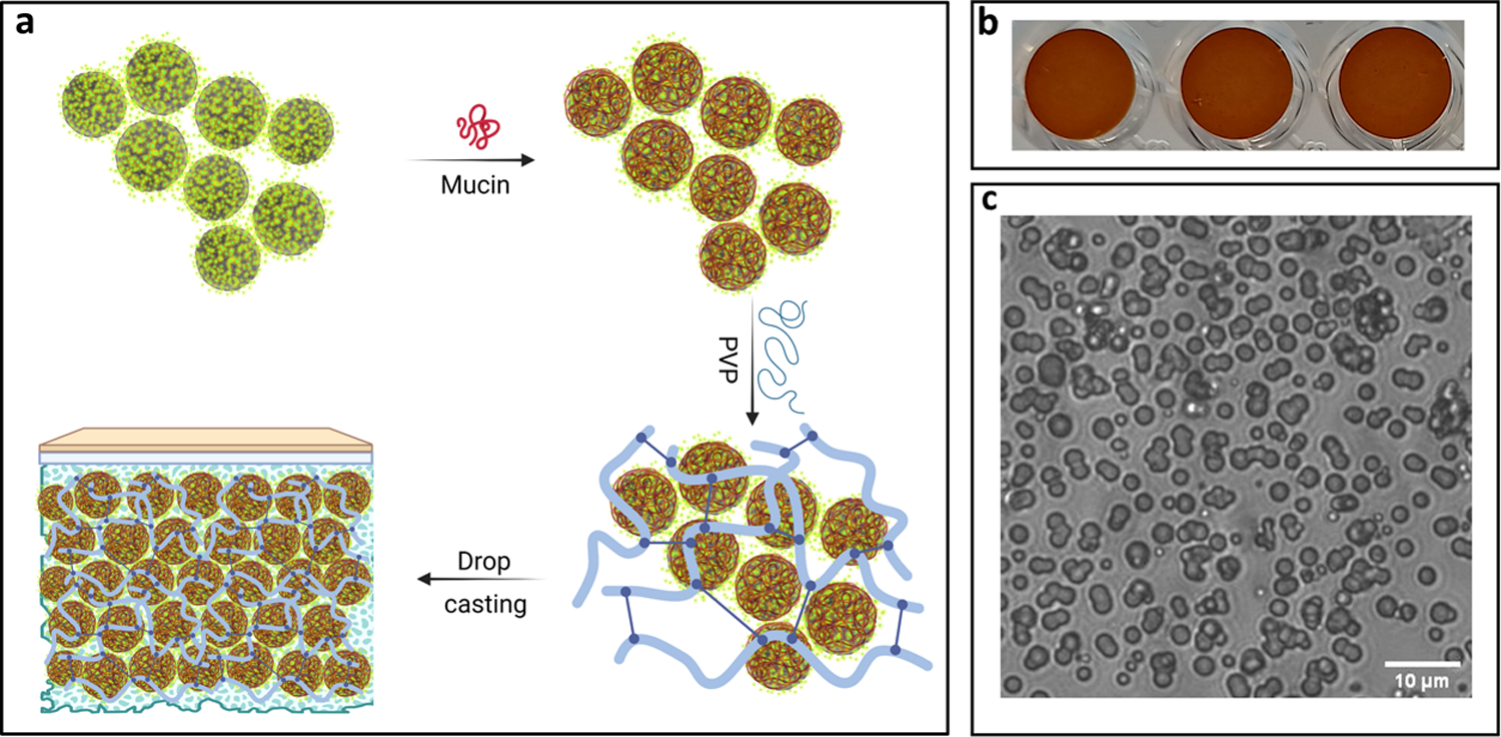
Schematic of coating B production method and expected structure after formation (a). Images b and c depict the coating B after drying and respective phase contrast light microscopy image, respectively. Scale bar is 10 μm for image c. Schematic created with BioRender.com.

Due to the quick recrystallization of vaterite, and consequent premature release of AgNPs, the CaCO_3_/AgNP crystals were precoated with mucin in an attempt to halt the transformation into calcite (Figure 2a). The precoated hybrids were then mixed with PVP as it improved the adhesion and uniformity of the coating.

As shown in Figure 2b, the formulation composed of hybrids, mucin, and PVP (coating B) resulted in uniform coatings. PVP in combination with mucin increased the viscosity and wettability of the formulation and consequently decreased the outflow and inflow of the hybrids. Moreover, precoating the hybrids with mucin prevented the crystal recrystallization, as shown by the transmittance image in Figure 2c, where the typical spherically shaped vaterite hybrids are visible. Overall, mucin not only formed a protective layer on the hybrids that suppressed the diffusion of ions and recrystallization but also contributed to the formation of uniform coatings. Song et al.^31^ have also reported that coatings composed of mucin present improved lubricity and reduced friction.

The formulation with PVP and mucin was selected to produce the final coatings with CaCO_3_/AgNP hybrids due to their uniformity and stability.

### 3.2 Characterization of the Coatings

After the formulation development and optimization, coating B was characterized by SEM, EDS, FTIR, and XRD to investigate the coating crystallinity, composition, and silver distribution.

**Figure 3:**
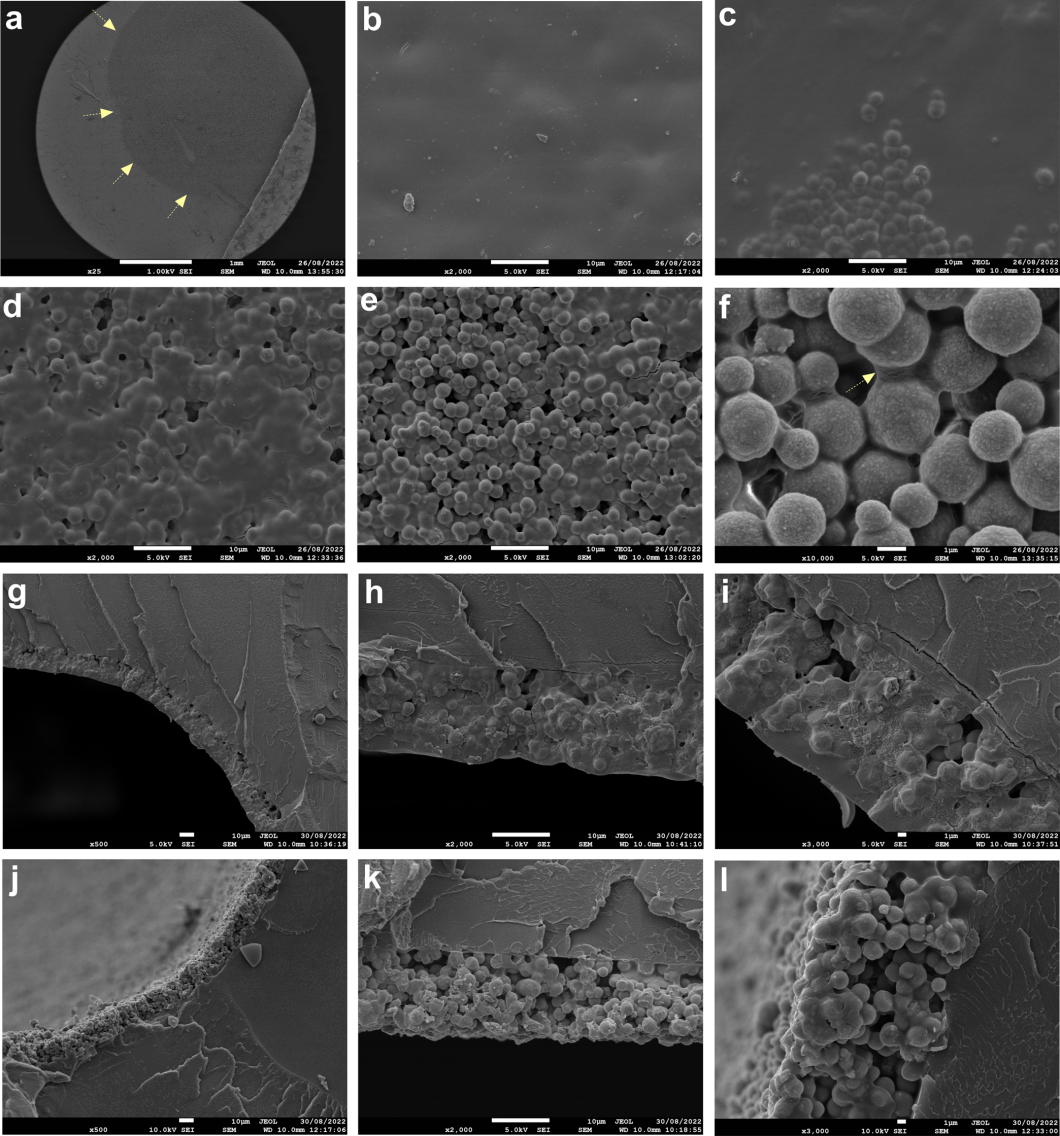
SEM images of the surface (a–f) and cross sections of the coatings (g–l). Image a: overview of coating B. Yellow arrows highlight the transition between the region with high (lighter gray) and low (darker gray) contents of PVP; images b–f: morphology of coating B surface in various regions gradually approaching the center. Images g–l: cross sections of the coatings in PVP-rich regions (g–i) and regions with lower PVP content (j–l). Scale bar represents 1 mm in image A, 10 μm in images b–e, g, h, j, and k, and 1 μm in images f, i, and l.

Figure 3a–f depicts SEM images of the surface of coating B. The coating presented an uneven distribution of PVP, with a gradient from the periphery to the center, with the highest contents of PVP being at the edges. This was reflected on the even surface in the regions rich in PVP (Figure 3b) that gradually became more coarse with proximity to the center (Figure 3c–f). The arrows in Figure 3a present the ring that marks the transition between the region with high (lighter gray) and low (darker gray) contents of PVP.

The irregular distribution of PVP seems to have been promoted by an evaporation-driven outflow of PVP to the periphery that packed the hybrids in the center. As PVP has a higher diffusion coefficient than the hybrids (larger radius), it diffused more rapidly to the edges of the well, accumulating there and consequently increasing the compactness of the hybrids in the center. Figure 3f shows that the center of the coated surface is composed of compacted hybrids connected to each other through polymer membranes (yellow arrow). Despite the nonuniform distribution of PVP, the thickness of the coatings was similar between replicates (15.2 ± 1.4 μm) and along the coating cross sections, regardless of the presence of more (Figure 3g–i) or less PVP (Figure 3j–l). These findings demonstrate the reproducibility of the produced coatings, nonetheless, with an irregular distribution of PVP.

Although not tested in this work, a potential solution to improve the distribution of PVP involves drying the coatings at room temperature rather than at 80 °C. This may decrease the rate of evaporation at the edges, thereby minimizing the outward flow of PVP to the periphery.

The images in Figure 3g–l demonstrate that the coatings are formed from multilayers of hybrids. Despite the random arrangement, the images indicate that the coatings consist of 6–7 layers of hybrids. This aligns with the theoretical prediction based on the diameter of the hybrids (2.3 ± 0.8 μm) and coating thickness (15.2 ± 1.4 μm). While each well presents a surface area of 0.29 cm^2^, it is estimated that after coating, the surface area increases exponentially due to the nanostructure of all of the components. This structure plays an important role in the performance of the coating. While not investigated in this study, this composite could potentially be used in the fabrication of customized scaffolds for tissue engineering purposes.

To analyze the elemental composition of the coatings and the silver distribution, the cross sections of the coatings were analyzed by SEM–EDS.

**Figure 4:**
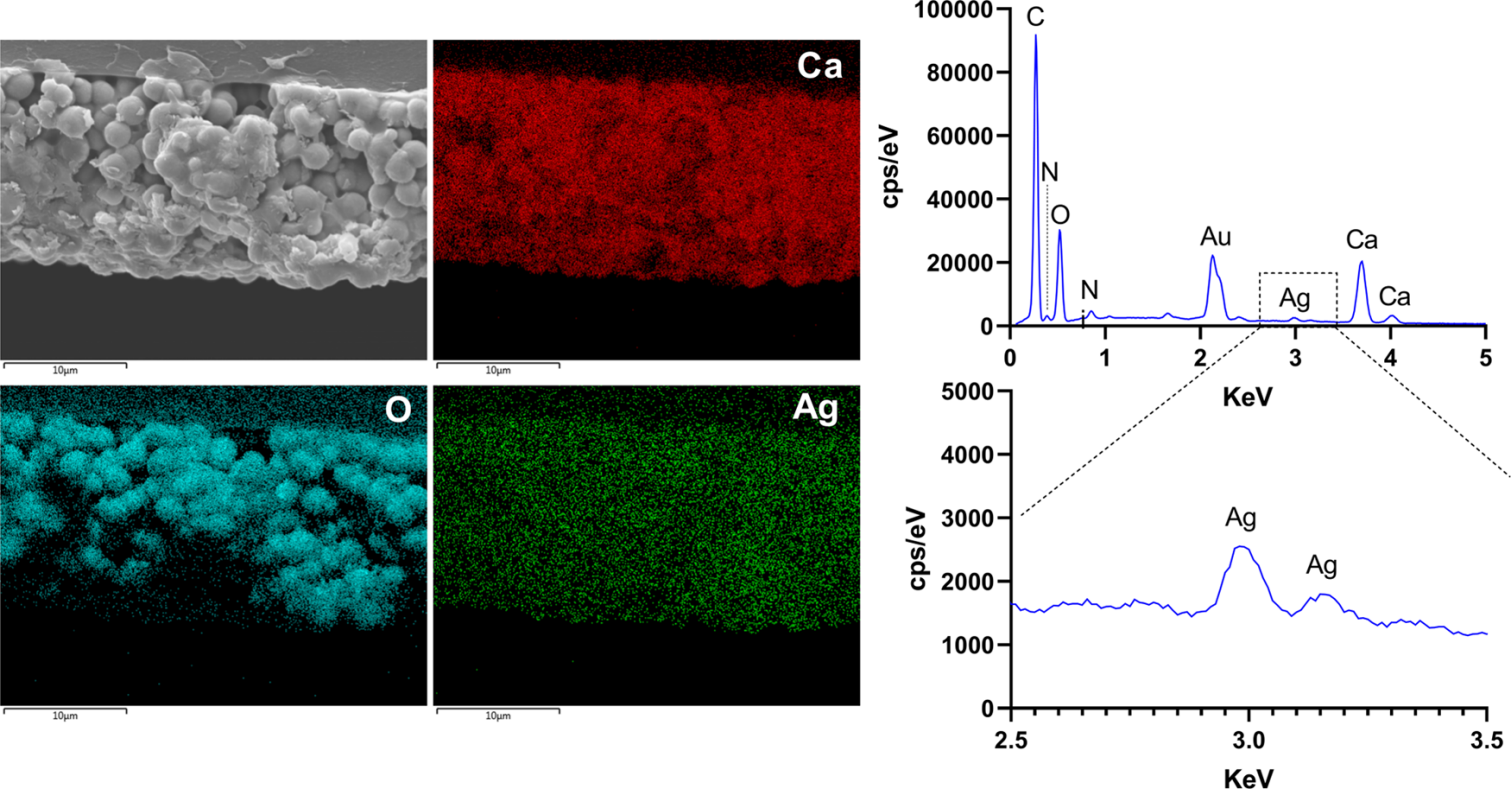
SEM–EDS mapping images and respective spectrogram of coating B.

Figure 4 depicts mapping images of calcium, oxygen, and silver and the spectrogram of the coating. As expected, the spectrogram demonstrates that the developed coating is mainly composed of carbon, oxygen, and calcium. Nitrogen and silver peaks resulting from the polymers (mucin and PVP) and AgNPs were also present, although the peaks presented lower intensities due to the low contents of nitrogen in PVP and mucin and silver in the hybrids (ca. 3%). The peak in the spectrogram assigned to gold resulted from the 5 nm layer of gold used to coat the samples for SEM–EDS analysis.

Overall, the mapping image of silver (Figure 4) shows that the AgNPs were uniformly distributed within the coating, which is an important feature for ensuring effective antimicrobial performance.

**Figure 5:**
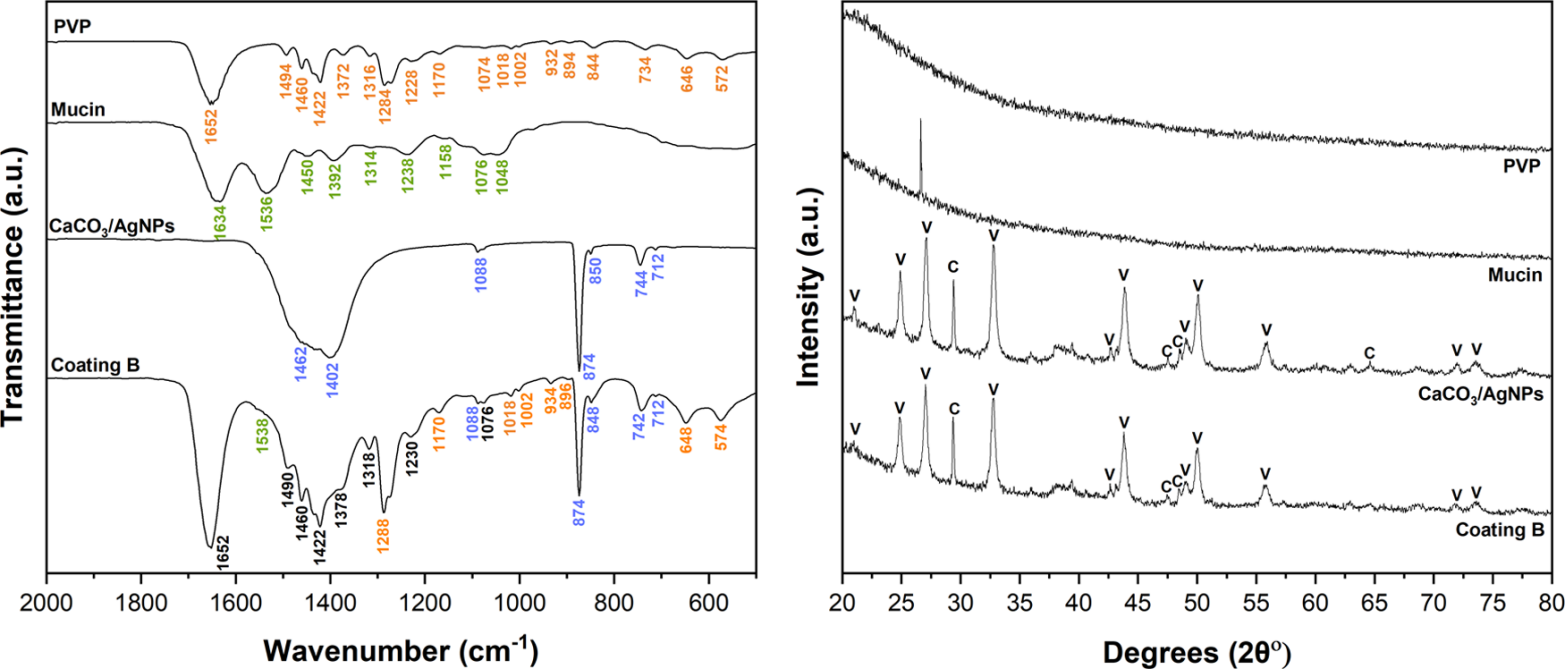
FTIR spectra (left) and XRD patterns (right) of PVP, mucin, CaCO_3_/AgNP hybrids, and coating B. The bands assigned in orange, green, and violet result from vibrational modes of PVP, mucin, and CaCO_3_/AgNP hybrids, respectively. The bands assigned in black in the FTIR spectra correspond to bands that resulted from the overlap of peaks from different compounds.

Figure 5 depicts the infrared spectra and XRD pattern of coating B and all of the constituents: PVP, mucin, and CaCO_3_/AgNP hybrids.

The FTIR spectrum of the coating presents the characteristic bands of all of the components (PVP, mucin, and CaCO_3_/AgNP hybrids). The typical bands of PVP, mucin, and CaCO_3_/AgNP hybrids are assigned in orange, green, and violet, respectively. The bands assigned in black indicate overlapping bands from multiple compounds.

The band at 1652 cm^–1^ resulted from the C=O stretching of the amide group present in PVP and mucin (amide I band).^32,33^ The low-intensity band at 1538 cm^–1^ resulted from N–H bending and C–N stretching vibrations of the proteins that compose mucin (amide II band).^32^ The region between 1490 and 1370 cm^–1^ resulted from an overlay of bands from all of the components in the coating, corresponding to C–H vibrations, antisymmetrical stretching, and vibration of the tertiary nitrogen and CO_3_^2–^.^13,32,34,35^ The other bands in the spectra resulted from amide group vibrations, O–P=O antisymmetric stretch, C–C, C–H, C–O, and C–O–H vibrations, with the last two resulting from carbohydrates present in mucin.^32^ The characteristic bands of CaCO_3_ were present at 1088, 874, 848, 742, and 712 cm^–1^. These bands resulted from vibrational modes of the CO_3_^2–^. The bands at 1088 and 742 cm^–1^ are characteristic of the vaterite polymorph, and the band at 712 cm^–1^ corresponds to calcite.^34,36^ This last band had a very low intensity, indicating the presence of low contents of calcite in the coating. These results show that vaterite is the main polymorph in coating B, which is corroborated by the SEM and transmittance images (Figures 2 and 3). The XRD data also showed that vaterite is the main polymorph (Figure 5, right), with the diffraction pattern of the coating presenting the typical peaks of vaterite and a few low intensity peaks assigned to calcite.

Overall, the data shows that mucin prevents the recrystallization of the hybrids into calcite, with the peaks assigned to CaCO_3_ in the FTIR spectra and XRD diffraction of both coating B and the hybrids being similar. This data demonstrates that during the coating production there was not a premature release of AgNPs from the hybrids triggered by their recrystallization into calcite. Ensuring that there is not an early release of AgNPs is crucial, as it can affect both the stability of the AgNPs and their antibacterial activity.

### 3.3 Release of AgNPs from the Coatings

The unwanted leaching of AgNPs or other active agents from antimicrobial coatings is undesirable. It decreases the antimicrobial activity of the surfaces and unnecessarily releases active ingredients into the environment that can have a toxic effect on living organisms and potentiate the appearance of antimicrobial resistance. In this work, the release of AgNPs from the developed coatings was studied in a TBS buffer and MHB.

The effect of mucin and PVP on the release of AgNPs was also analyzed by testing the coatings composed of only hybrids (coating A) and the coatings composed of hybrids, PVP, and mucin (coating B).

**Figure 6:**
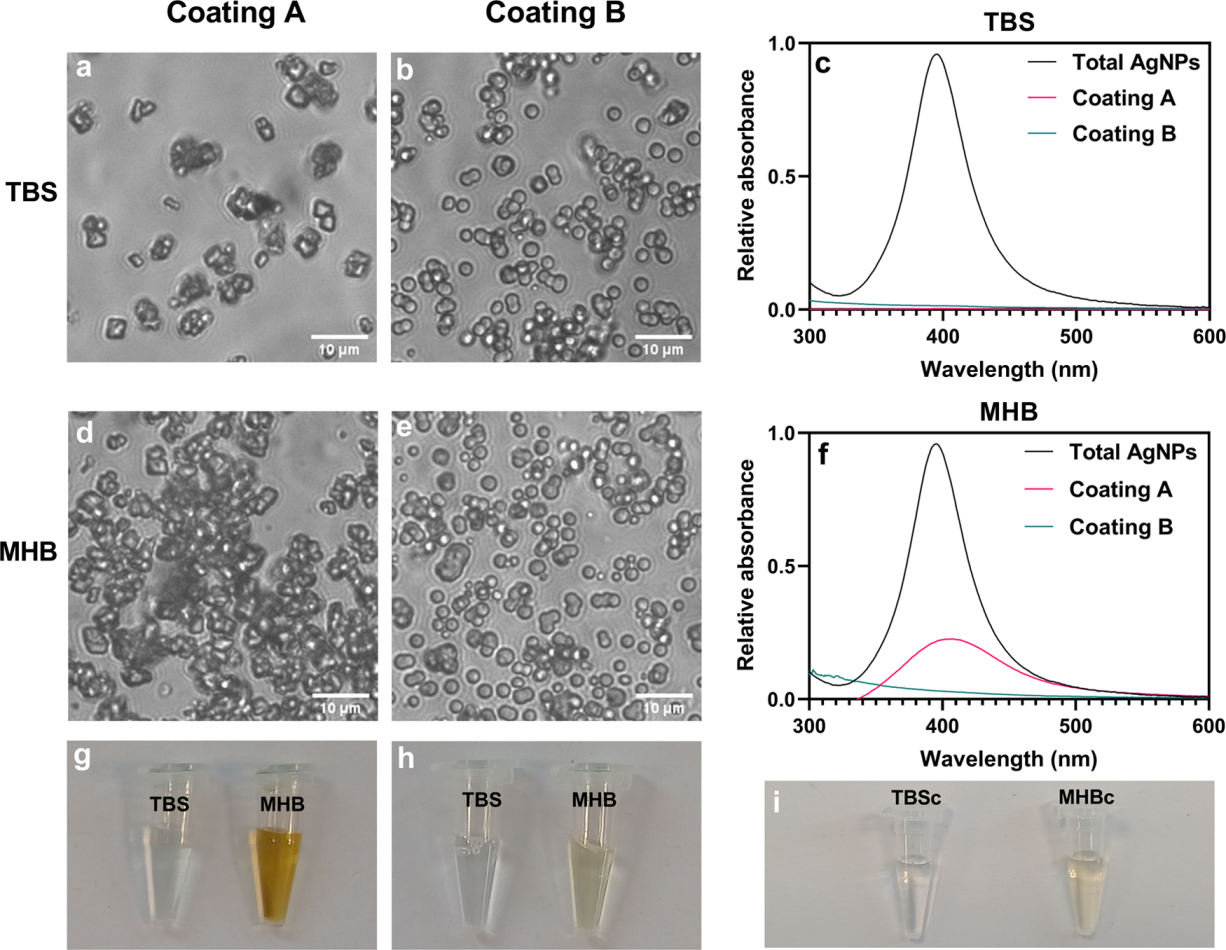
Phase contrast transmitted light microscopy images of the coating only composed of hybrids (coating A) and composed of hybrids, PVP, and mucin (coating B) after being exposed for ca. 19 h to TBS (a, b) and MHB (d and e). Images g and h present the supernatant of TBS and MHB after incubation with coating A and coating B, respectively. The TBS and MHB controls, TBSc and MHBc, respectively, are presented in Image i. Graphs c and f present the UV–vis spectra of MHB and TBS after incubation with coating A and coating B. The control spectrum corresponds to the equivalent concentration of the total content AgNPs (total AgNPs) in the coatings.

Figure 6a,b,d,e depicts the transmittance images of the coatings after being exposed to TBS and MHB. As expected, the coatings without PVP and mucin were composed of calcite (cubic shape), as the hybrids recrystallized during the coating production. On the other hand, the coatings with PVP and mucin did not recrystallize even after incubation in TBS and MHB for approximately 19 h, highlighting the good stabilizing properties of mucin.

Figure 6c,f presents the UV–vis spectra of TBS and MHB after incubation with the coatings. UV–vis spectrophotometry is a good method to detect the presence of AgNPs and their concentration, as it is highly sensitive to AgNPs due to their surface plasmon-resonance property.^37−39^

Interestingly, none of the coatings released AgNPs in TBS. While this would be more expected for the coatings with additives, as the hybrids did not recrystallize, the same was not expected for coating A. This result can be attributed to calcite adsorbing AgNPs and therefore retaining the AgNPs within the coating. The capacity of calcite to adsorb AgNPs after recrystallization has been demonstrated before by our research group and was shown to be controlled by the affinity between the AgNPs and CaCO_3_, as well as the medium composition.^4,40^ When the medium is rich in biomolecules that adsorb to AgNPs, the affinity to CaCO_3_ decreases, and the release of the nanoparticles is triggered. This effect has been reported before by our research group^40^ and is demonstrated in this work by the partial release of AgNPs from coating A in MHB as shown by the UV–vis results (Figure 6f) and the color of MHB solution after incubation with coating A (Figure 6h). AgNPs are visually detectable due to their characteristic amber color. As MHB is rich in biomolecules such as casein and starch, it triggers the partial release of AgNPs by decreasing their affinity to CaCO_3_. On the other hand, coating B did not release AgNPs in MHB, as shown by the image of MHB after incubation (Figure 6g) and the UV–vis results (Figure 6c). These results show the crucial role of mucin in preventing interactions between the AgNPs loaded into the hybrids and MHB components, as well as inhibiting the transformation of vaterite into calcite. Due to the high content of PVP in the coating, it is also expected that PVP plays an important role in preventing the release of AgNPs.

Previous work published by our research group demonstrated that the release of AgNPs from CaCO_3_/AgNP hybrids is pH-dependent.^4,40^ Based on these results, coating B presents the potential to be used in applications where the release of AgNPs is desired at low pH values, such as the core of biofilms and sites of acute inflammation. The low pH in these microenvironments^7,41^ can promote the dissolution of CaCO_3_ and consequent release of AgNPs.

### 3.4 Antibacterial Activity

The antibacterial activity of the coatings was assessed using the resazurin reduction assay. This method consists of determining bacterial viability by measuring the fluorescence of resorufin, a molecule produced via the reduction of resazurin by viable bacteria. Before testing the coatings, the applicability of this method in assessing the viability of *E. coli*, MRSA, and *P. aeruginosa* was studied by incubating different concentrations of bacteria with resazurin. Figure S2 presents the developed fluorescence after incubation with different concentrations of bacteria, expressed as increasing optical density values (OD_600_). The data showed that this assay is sensitive to the concentration of bacteria with the fluorescence increasing linearly with the concentration of bacteria. The resazurin assay, coupled with the microplate coating layout proposed in this work, enables a rapid assessment of the antibacterial activity of multiple coatings and their replicates. Furthermore, it outpaces more laborious methods such as those reported in JIS Z 2801^42^ and ISO 22196^43^ standards.

The antibacterial activity of coatings A and B was tested to study the effect of PVP and mucin in the overall activity of the coatings. Figure 8 presents the relative fluorescence for *E. coli*, MRSA, and *P. aeruginosa* after incubation with the coatings containing different concentrations of hybrids. Overall, the coatings with PVP and mucin presented better antibacterial activity than those composed solely of hybrids. Coating B inhibited the growth of *E. coli* and *P. aeruginosa* at a density of 15 μg/cm^2^ and MRSA at twice this density, *i.e*., 29 μg/cm^2^. Coating A required higher concentrations of hybrids to inhibit the growth of the bacteria, inhibiting *E. coli* and MRSA growth at densities of 233 and 931 μg/cm^2^, respectively. The lowest concentration of hybrids in coating A tested against *P. aeruginosa* inhibited the bacteria effectively, demonstrating that the minimum concentration with bactericidal activity against *P. aeruginosa* is below 58 μg/cm^2^.

To confirm the complete eradication of the bacteria by coating B, aliquots were spot-plated onto MHA plates after the resazurin assay. As shown in Figure S7, the density of hybrids with biocidal activity was 15 μg/cm^2^ for *P. aeruginosa* and 29 μg/cm^2^ for *E. coli* and MRSA. The results matched the data in Figure 8, except for *E. coli*, which presented viable bacteria in one of the replicates tested at 15 μg/cm^2^. Based on these results, the density of AgNPs in coating B with bactericidal activity was 0.9 and 0.4 μg/cm^2^, which corresponds to the coatings with 29 and 15 μg/cm^2^ of hybrids, respectively.

The low concentrations of AgNPs needed to kill the bacteria demonstrate that the coatings have strong cytotoxicity against *E. coli*, MRSA, and *P. aeruginosa*, three bacteria responsible for numerous serious and life-threatening infections in hospital settings.

The toxicity mechanisms of AgNPs are not fully understood, but it is believed that the release of Ag^+^ from the nanoparticles is crucial to kill the bacteria. Ag^+^ can disrupt the membrane, and once uptaken by the cell, it can interact with disulfide or sulfhydryl groups of intracellular enzymes, leading to the disruption of metabolic processes like adenosine triphosphate release and increasing the production of reactive oxygen species.^44−46^ Additionally, Ag^+^ can interact with the DNA, disrupting DNA replication and cell propagation, and denature the cytoplasmic ribosomal components hindering protein synthesis.^44−46^ It is believed that the AgNPs also play a role in killing the bacteria through denaturation of the membrane and modification of the cell wall structure, which can lead to leakage of cellular contents and cell death.^44−46^ Nonetheless, Xiu et al.^47^ have shown that the antibacterial activity of AgNPs against *E. coli* depends mainly on Ag^+^ release, highlighting the role of the ions over the particles in eradicating the bacteria.

**Figure 7:**
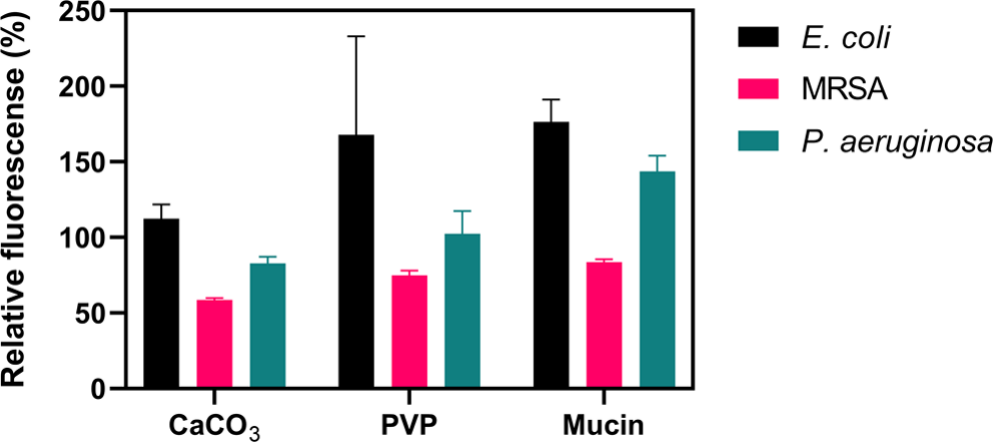
Developed relative fluorescence after incubation of the control coatings with bacteria for 24 h. The control coatings were composed of unloaded CaCO_3_, PVP or mucin. The fluorescence is relative to the growth control. The results represent an average of four or five replicates.

**Figure 8:**
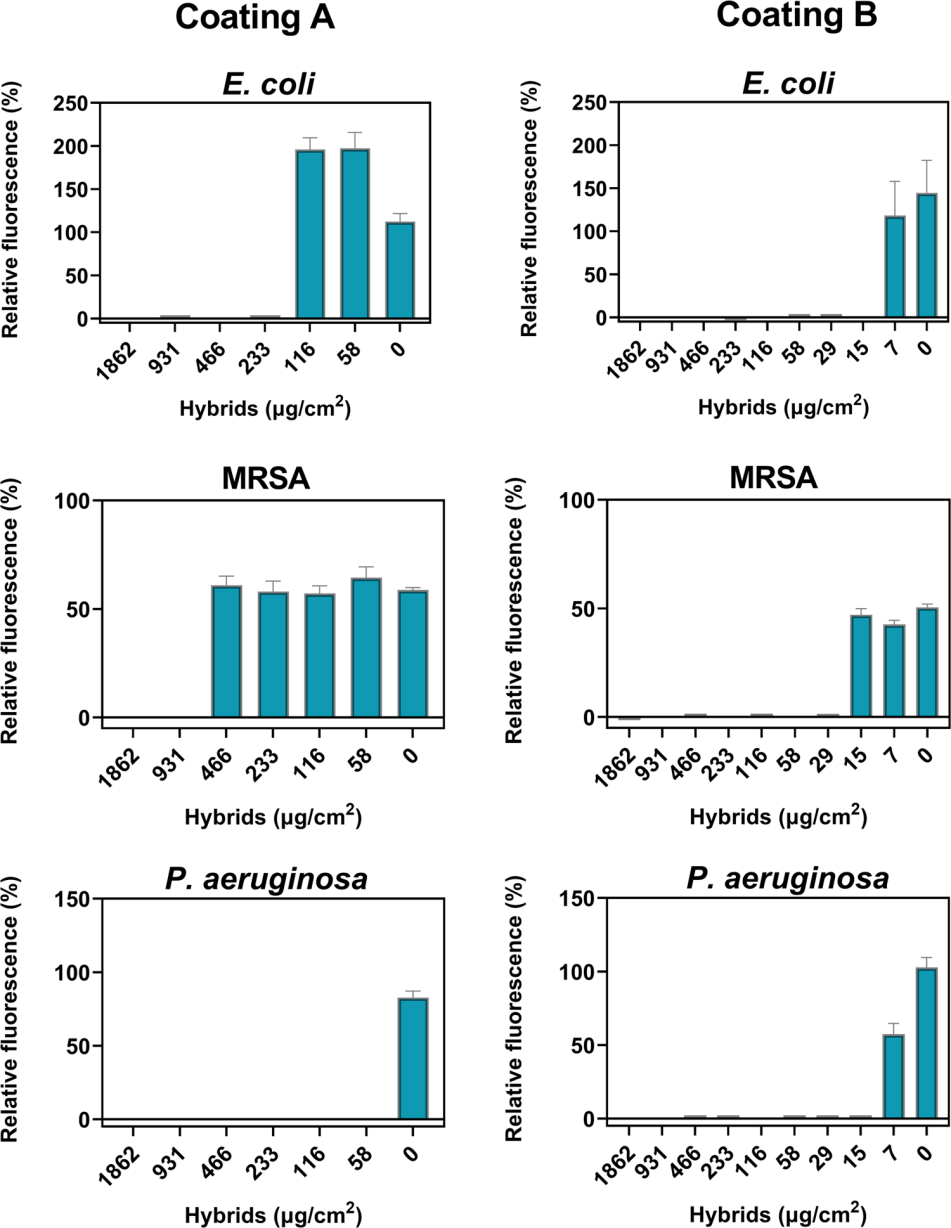
Relative fluorescence after incubation of the bacteria with the coatings for 24 h. The data corresponds to the coatings composed of hybrids (coating A) and hybrids, mucin, and PVP (coating B) at different concentrations of the CaCO_3_/AgNP hybrids. The fluorescence is relative to the growth control. The results present the average of four or five replicates.

To analyze the effect of PVP, mucin, and bare CaCO_3_ against the bacteria, coatings with these components were produced and their bacterial cytotoxicity was assessed. Figure 7 presents the relative fluorescence after incubation. The data shows that PVP and mucin do not eradicate the bacteria, with PVP even promoting the growth of *E. coli*, possibly by increasing the hydration of the bacteria.^48^ Mucin also promoted the growth of *E. coli* and *P. aeruginosa* as it can work as an additional energy source for these bacteria.^49−51^ In the case of unloaded CaCO_3_, it did not considerably affect the growth of *E. coli* and *P. aeruginosa*. On the other hand, it promoted a reduction in the number of viable MRSA bacteria (41% less than the control). This was also evidenced in the tests carried out with coatings A and B against MRSA (Figure 8), where the coatings without bactericidal activity reduced the growth of MRSA to around 60%.

It has been demonstrated by Xie et al.^52^ that calcium ions (Ca^2+^) can kill *Staphylococcus aureus* (*S. aureus*) by promoting the destabilization of the membrane via the formation of complexes with cardiolipin (CL), a major lipid component in *S. aureus* membranes. The same researchers also demonstrated that Ca^2+^ did not present activity against *E. coli* due to the low content of CL on their membranes. The same trend has also been reported by Thanakkasaranee et al.^53^ These findings explain the results obtained in this work. CaCO_3_ worked as a source of Ca^2+^, which then interacted with the CL present in the membrane of MRSA, disrupting the bacteria.

The bactericidal results for unloaded CaCO_3_, mucin, and PVP demonstrate that the difference between the bactericidal activity of coatings A and B is not caused by PVP, mucin, or bare CaCO_3_, as the first two do not have bactericidal activity, and unloaded CaCO_3_ was present at equal concentrations in both coatings. The enhanced bactericidal activity of the coatings with PVP and mucin, 8–32 times greater against *E. coli* and MRSA, respectively, seems to have been promoted by the structure and uniformity of the coatings (Figure S6). This resulted in an even distribution of AgNPs, the main component with a bactericidal effect. Moreover, the coatings with PVP and mucin stopped the recrystallization of the hybrids and premature release of AgNPs. This helped to prevent the formation of AgNP clusters during the drying step at 80 °C. As shown in Figure S6, the coating without PVP and mucin presented darker colors, an indicator of AgNP agglomeration. One of the major problems associated with AgNP agglomeration is the reduction of the surface area, which decreases the release of silver ions (Ag^+^), crucial for the bactericidal activity of AgNPs.^47^

Overall, the results demonstrate that mucin and PVP played a pivotal role in preserving the nanostructure of the coatings by inhibiting the transformation of vaterite into calcite and agglomeration of the AgNPs. This resulted in a composite with a larger surface area and enhanced functionality.

**Figure 9:**
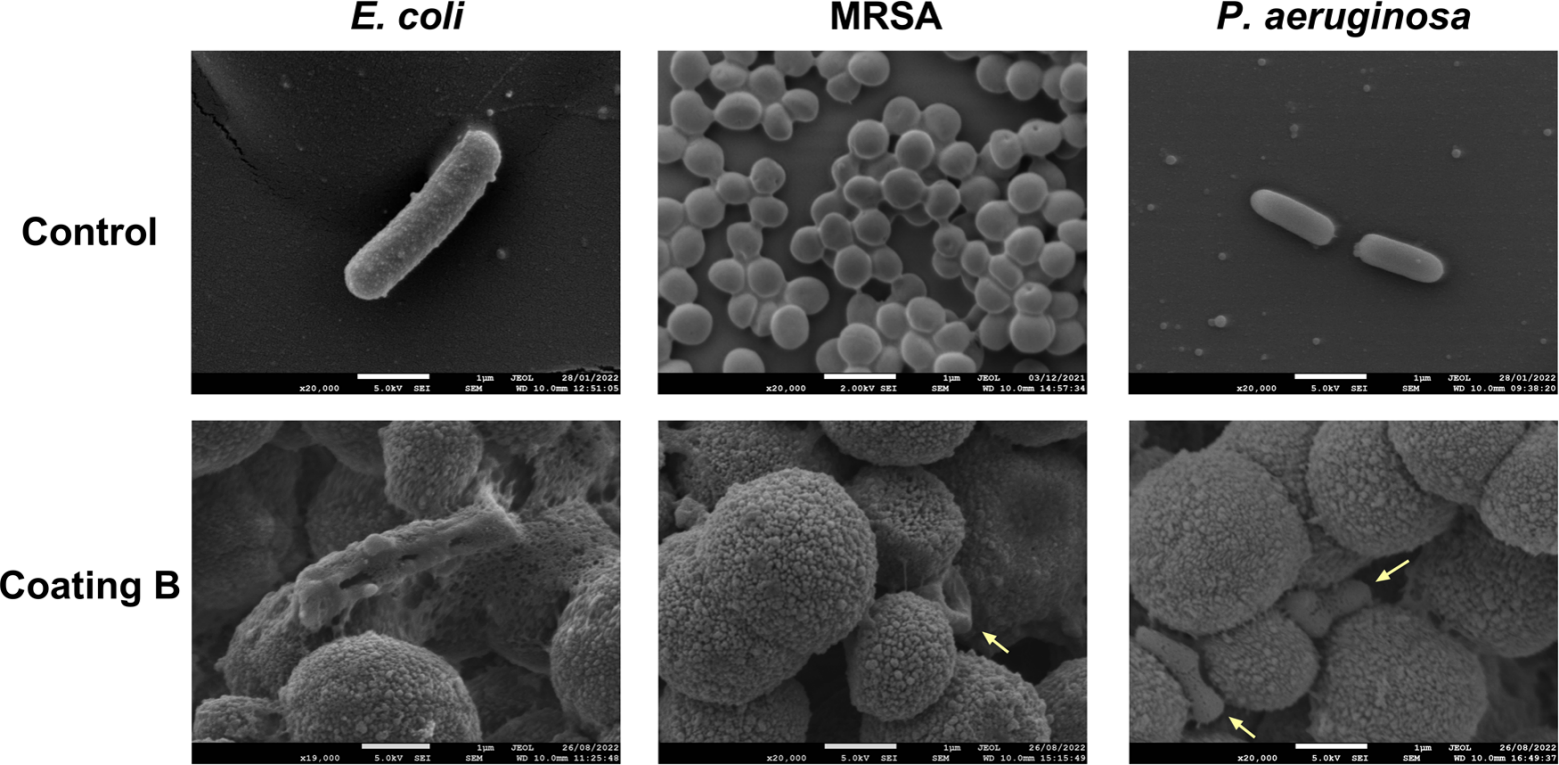
SEM images of *E. coli*, MRSA, and *P. aeruginosa* before (Control) and after (Coating B) being exposed to coating B at 23 μg/cm^2^ and left for 2 h.

Figure 9 depicts the SEM images of coating B (29 μg/cm^2^) after incubation with *E. coli*, MRSA, and *P. aeruginosa* for 2 h and the respective controls.

The bacteria in the control samples presented a well-preserved cell membrane without any damage or cell deformation. Nonetheless, after contact with the coated surface, the bacteria presented severely damaged and perforated membranes. These results are in agreement with previous reports and demonstrate that the toxicity mechanism of the AgNPs includes the disruption of the cell membrane.^54−56^ Regardless of the bacterial type, no cells with intact membranes were observed on the coated surfaces, showing that the coating exerted its bactericidal activity within 2 h of contact with the bacteria.

### 3.5 *In Vitro* Cytotoxicity

**Figure 10:**
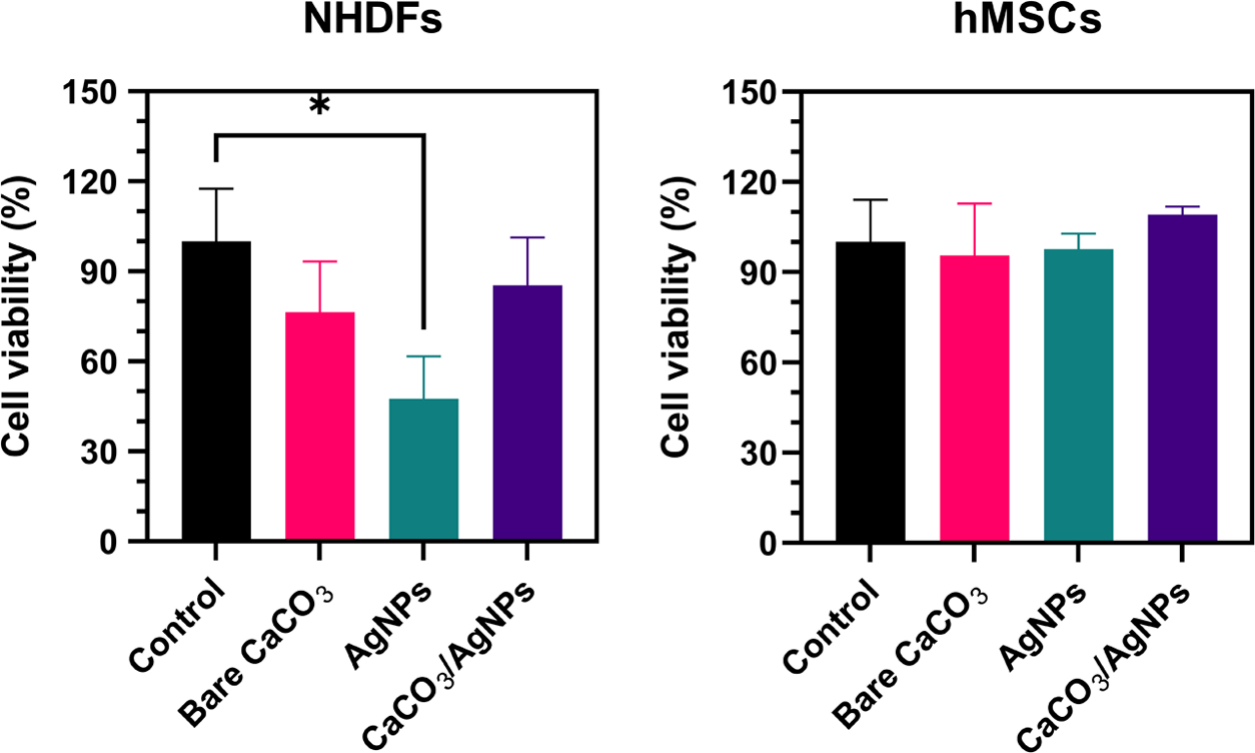
NHDFs and hMSC cell viability after incubation for 24 h with CaCO_3_/AgNP hybrids with bactericidal activity and the equivalent concentration of unloaded CaCO_3_ and AgNPs. Statistical analysis was made using the Kruskal–Wallis test/Dunn’s post-test (*p < 0.05 vs control). The results are an average of three or four replicates.

While the aim was to develop a coating with high toxicity against bacteria, it was important to ensure that it did not pose toxicity to human cells at bactericidal concentrations. To evaluate the toxicity effect of the hybrids on human cells, NHDFs and hMSCs were exposed to the same concentrations of hybrids that showed bactericidal activity (29 μg/cm^2^). The equivalent concentrations of unloaded CaCO_3_ and AgNPs were also tested. As shown in Figure 10, the hybrids did not significantly affect the viability of either cell line or the unloaded CaCO_3_. On the other hand, while the equivalent concentration of AgNPs did not affect the viability of hMSCs, they reduced the survival of NHDFs. The enhanced resistance of hMSCs to lethal compounds has previously been demonstrated, with studies showing that hMSCs are highly resistant to apoptosis after exposure to DNA-damaging agents, with senescence being one of the mechanisms to evade drug-induced apoptosis.^57^ The capacity of hMSCs to evade toxic compounds explains their survival when exposed to AgNPs. In the case of NHDFs, the hybrids decreased the toxicity of the AgNPs. As the AgNPs are immobilized on CaCO_3_, the interactions between AgNPs and the cells are minimized, and therefore the toxicity of the AgNPs was decreased. Phase contrast transmitted light microscopy images of the cells after incubation with the hybrids, unloaded CaCO_3_, and AgNPs are presented in Figure S8.

Overall, these results evidenced that the hybrids did not affect the viability of human cells at concentrations used to produce a bactericidal effect. Future work should focus on determining the effect of the coating formulation on mammalian cells and on *in vivo* studies.

## 4 Conclusions

Coatings composed of CaCO_3_/AgNP hybrids, with approximately 3% (w/w) of loaded AgNPs, were developed. The production steps were optimized, and a formulation consisting of hybrids, PVP, and mucin developed. The optimized formulation resulted in macroscopically uniform coatings, with mucin playing a crucial role in preventing the recrystallization of the hybrids into calcite and the consequent release of AgNPs. Further characterization of the coating showed that PVP accumulated more in the periphery and the hybrids in the centre. Nonetheless, all the coatings presented a uniform thickness, 15 μm, which was reproducible between replicates. The SEM–EDS analysis showed that AgNPs were homogeneously distributed in the coating, and the XRD and FTIR analysis proved that mucin prevented the recrystallization of the hybrids, with the main polymorph in the coating being vaterite. The release of AgNPs from the coatings was tested. The results showed that the coatings with PVP and mucin did not release AgNPs, an important feature in preventing the unwanted release of the active component. The antibacterial studies of the coatings showed that PVP and mucin play an important role in enhancing the bactericidal activity by improving the uniformity of the coating and halting the recrystallization of the hybrids and consequent premature release of AgNPs. The coating presented a strong bactericidal activity against *E. coli*, MRSA, and *P. aeruginosa*, killing the bacteria at hybrid concentrations between 15 and 29 μg/cm^2^. The bactericidal tests also demonstrated that Ca^2+^ can reduce the proliferation rate of MRSA bacteria. The *in vitro* cytotoxicity tests showed that the hybrids at bactericidal concentrations do not affect the viability of human cells. Overall, the coating developed in this work demonstrated the potential of CaCO_3_/AgNP hybrids for the production of surfaces with bactericidal properties, as long as their premature recrystallization is halted. The methods proposed in this work for the rapid production of coatings and straightforward assessment of their bactericidal activity present new alternatives to accelerate the analysis of materials with bactericidal activity. Future work should focus on evaluating the potential of the developed coating in eradicating bacteria through photothermal therapy as well as test their biocompatibility *in vivo* and explore the association of the hybrids with other active compounds.

## Supporting Information Available

Scheme of the coatings in the 96-well microplate, resorufin assay correlation with the bacterial concentration, TEM image of the AgNPs, images of the coatings produced at different conditions, spot plating of the bacterial inoculum after incubation with the coatings, and transmittance images of the NHDFs and hMSC cells after being exposed to the crystals (PDF)

## Abbreviations

AgNPs: silver nanoparticles
CaCO_3_: calcium carbonate
CACs: composite antimicrobial coatings
Coating A: CaCO_3_/AgNP hybrids
Coating B: CaCO_3_/AgNP hybrids, mucin, and PVP
PVP: polyvinylpyrrolidone
