# Supplementary material for: Nanoarchitectonics of Bactericidal Coatings Based on CaCO3–Nanosilver Hybrids
Source: ACS Appl Bio Mater. 2024 May 9;7(5):2872–86. doi: 10.1021/acsabm.3c01228 (PMC11110054; doi:10.1021/acsabm.3c01228)
Supplement: Supplementary file 1 — mt3c01228_si_001.pdf [file mt3c01228_si_001.pdf]

Supporting Information

Nanoarchitectonics of Bactericidal Coatings Based on $CaCO_3$-Nanosilver Hybrids

Ana M. Ferreira[1], *Anna Vikulina*[1,2], *Laura Bowker*[1], *John A. Hunt*[1], *Michael Loughlin*[1], *Valeria Puddu*[1], *Dmitry Volodkin*[1*]

[1]School of Science and Technology, Department of Chemistry and Forensics, Nottingham Trent University, Clifton Lane, Nottingham NG11 8NS, UK.

[2]Bavarian Polymer Institute, Friedrich-Alexander-Universität Erlangen-Nürnberg (FAU), Dr.-Mack-Straße, 77, 90762 Fürth, Germany

Corresponding author*: Dmitry Volodkin, E-mail: dmitry.volodkin@ntu.ac.uk

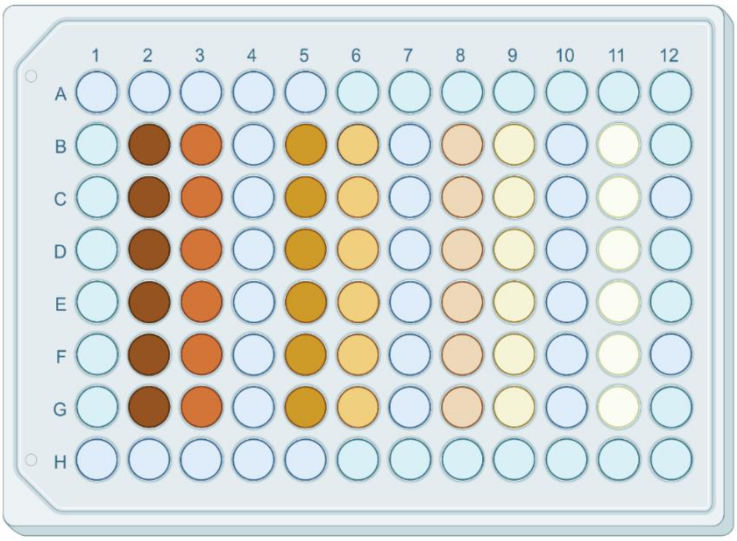

Figure S1. Schematic of the coatings in the 96-well microplate. The brown/yellow wells represent the wells coated with different concentrations of hybrids, with the dark brown wells representing the coatings with the highest concentrations of hybrids. The blue wells represent the wells that were filled with PBS to prevent the dehydration of the bacteria. Created with BioRender.com

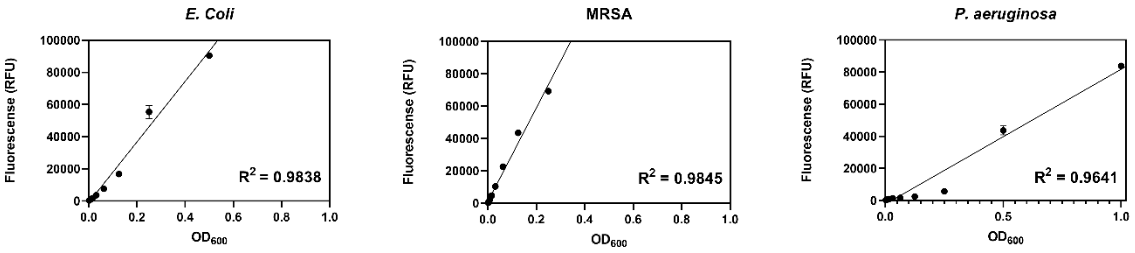

Figure S2. Resorufin fluorescence versus the bacterial concentration represented by increasing optical densities at 600 nm (OD_{600})

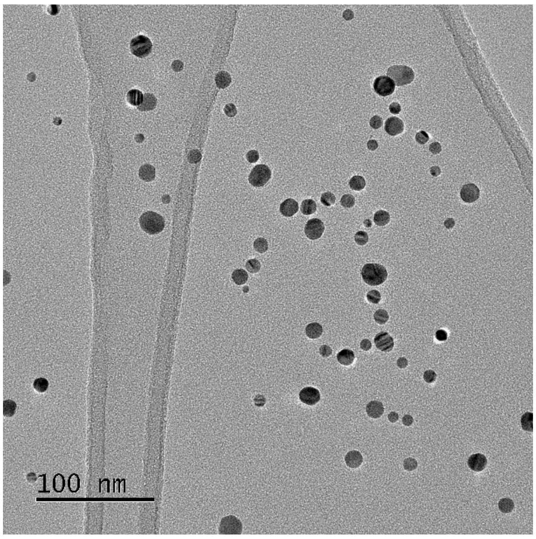

Figure S3. TEM image of the AgNPs

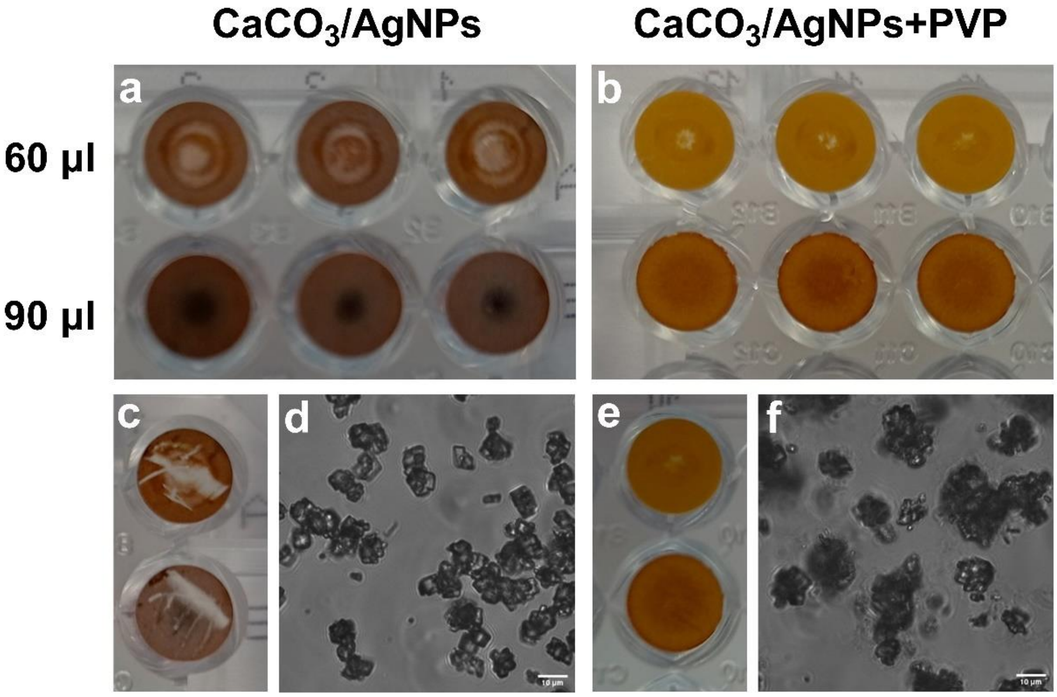

Figure S4. Wells coated with different volumes (60 and 90 µl) of $CaCO_3$/AgNPs hybrids (Hybrids) or $CaCO_3$/AgNPs hybrids with PVP (Hybrids + PVP) by drop casting (image a and

b), and coatings after the scratch test (image c and e). The phase contrast transmitted light microscopy images of the coatings after drying at a 50/37°C for 2 and 20h, respectively, are presented in image d and f. Scale bar in images d and f represents 10 µm

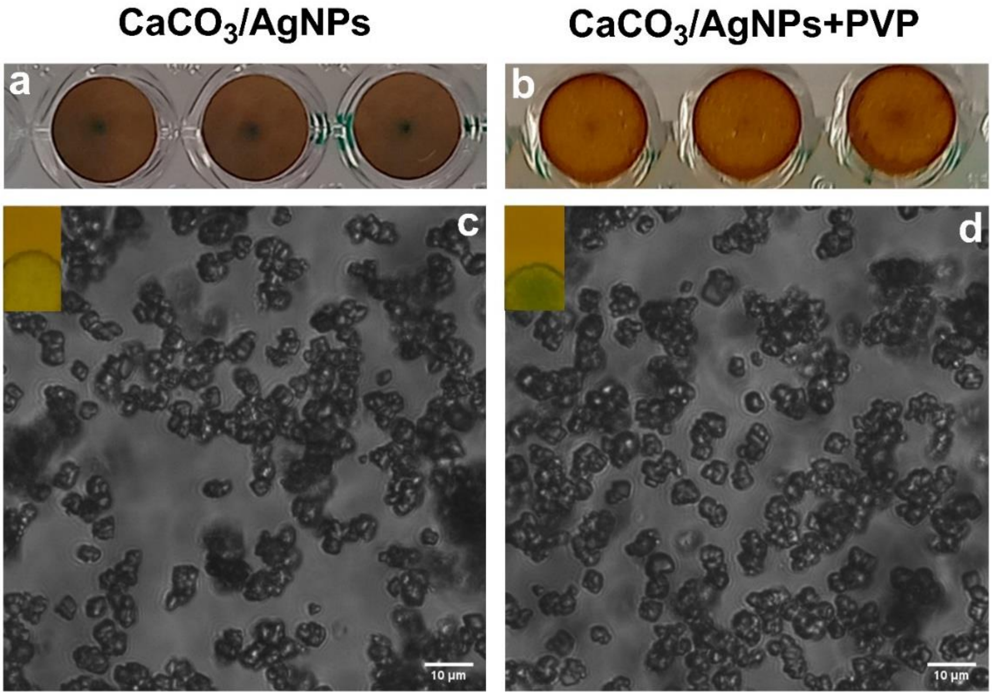

Figure S5. Images a and b present the wells coated by drop casting with 90 µl of $CaCO_3$/AgNPs dispersion (a) and $CaCO_3$/AgNPs hybrids with PVP (Hybrids + PVP, b) . Images c and d depict phase contrast transmitted light microscopy images of the coatings after drying at 80°C (2 h). Insets in the transmission images correspond to the universal indicator tape (pH 1-14) after dipping into the dispersions. Scale bar represents 10 µm in Images c and d

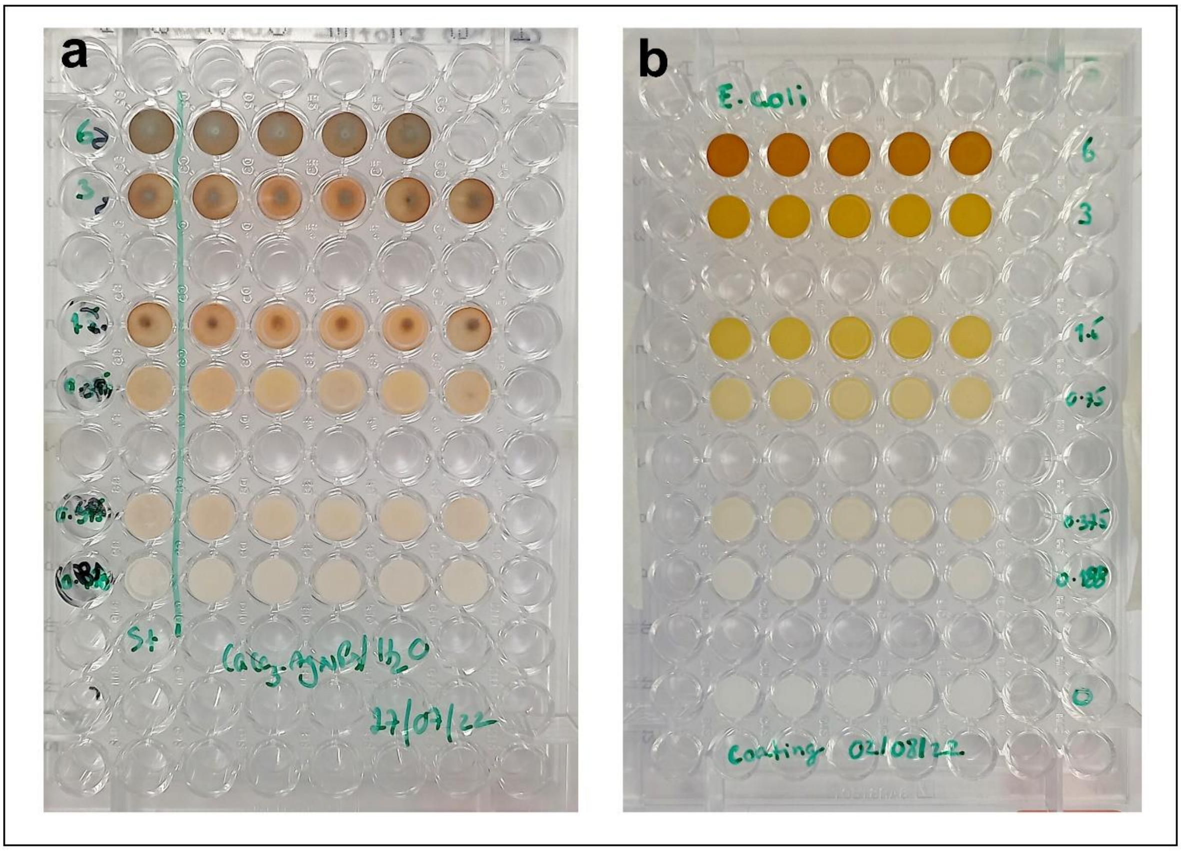

Figure S6. Example of microplates coated with different concentrations of $CaCO_3$/AgNPs hybrids (coating A, image a) and $CaCO_3$/AgNPs hybrids with the additives mucin and PVP (coating B, image b)

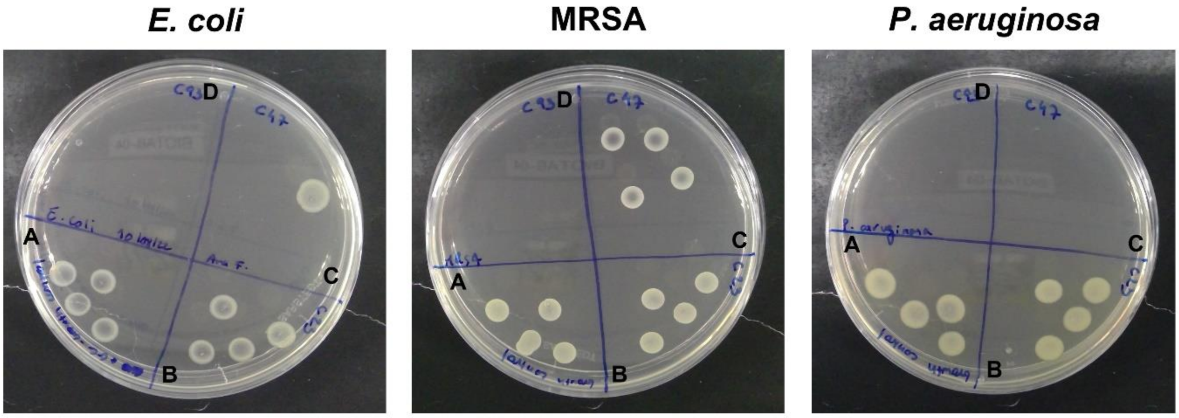

Figure S7. Spot plating of the bacterial inoculum after incubation with coating B. The growth controls of the bacteria incubated on uncoated wells are represented by the letter A. The coatings presented the following hybrids densities: 7, 15 and 29 μg/cm^2, which are represented by the letters B, C and D, respectively. The labels 23, 47 and 93 in the plate are respective to the concentration of the hybrids (μg.ml^{-1}) in the dispersions used to produce the coatings, which resulted in densities equal to 7, 15 and 29 μg/cm^2, respectively

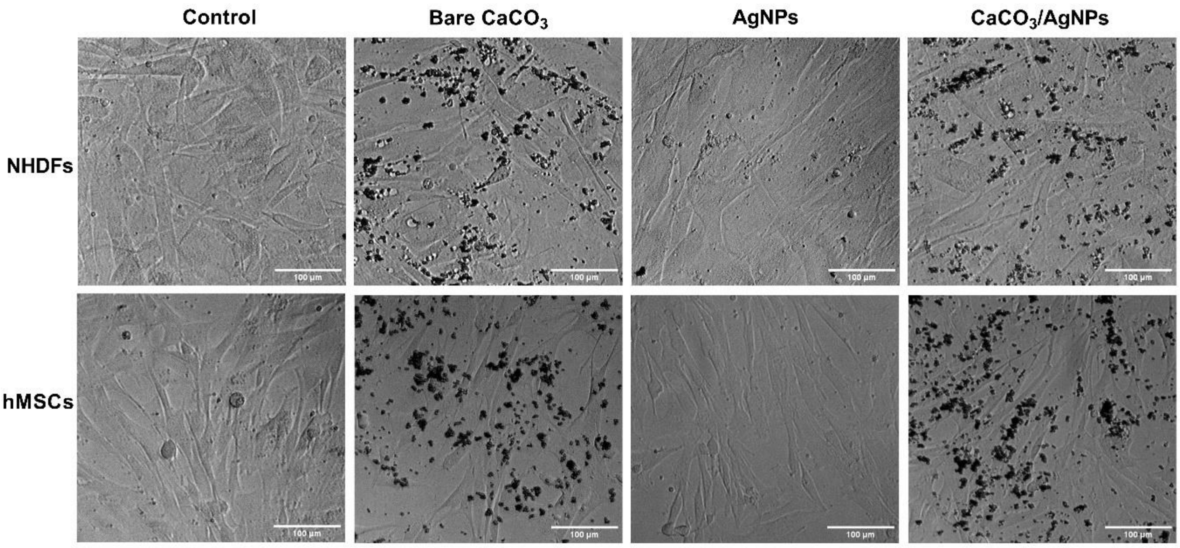

Figure S8. Phase contrast transmitted light microscopy images of the NHDFs and hMSCs cells after being exposed for 24 h to $CaCO_3$/AgNPs hybrids (29 μg/cm^2) and the equivalent

concentration of bare $CaCO_3$ (29 μg/cm^2) and AgNPs (0.87 μg/cm^2). Dark regions in the images correspond to the hybrids.
